# The Cost-Effectiveness of HIV/STI Prevention in High-Income Countries with Concentrated Epidemic Settings: A Scoping Review

**DOI:** 10.1007/s10461-022-03583-y

**Published:** 2022-01-15

**Authors:** Palmo Brunner, Karma Brunner, Daniel Kübler

**Affiliations:** Department of Political Science, University of Zurich, Affolternstrasse 56, 8050 Zurich, Switzerland

**Keywords:** HIV/STI prevention, Economic evaluations, Scoping review, Prevención del VIH/ITS, evaluaciones económicas, revision de alcance

## Abstract

The purpose of this scoping review is to establish the state of the art on economic evaluations in the field of HIV/STI prevention in high-income countries with concentrated epidemic settings and to assess what we know about the cost-effectiveness of different measures. We reviewed economic evaluations of HIV/STI prevention measures published in the Web of Science and Cost-Effectiveness Registry databases. We included a total of 157 studies focusing on structural, behavioural, and biomedical interventions, covering a variety of contexts, target populations and approaches. The majority of studies are based on mathematical modelling and demonstrate that the preventive measures under scrutiny are cost-effective. Interventions targeted at high-risk populations yield the most favourable results. The generalisability and transferability of the study results are limited due to the heterogeneity of the populations, settings and methods involved. Furthermore, the results depend heavily on modelling assumptions. Since evidence is unequally distributed, we discuss implications for future research.

**Supplementary Information:**

The online version contains supplementary material available at 10.1007/s10461-022-03583-y.

## Resumen

El objetivo de esta revisión de alcance (“scoping review”) es exeminar las investigaciones realizadas acerca de las evaluaciones económicas en el campo de la prevención del VIH/ITS en países de altos ingresos con epidemia concentrada y evaluar actuales conocimientos sobre las relaciones costoeficacia de las diferentes medidas. Con este objetivo han sido revi-sadas las evaluaciones económicas de las medidas de prevención del VIH/ITS publicadas en las bases de datos Web of Science y Cost- Effectiveness Registry. Incluidos fueron 157 estudios sobre intervenciones estructurales, conductuales y biomédicas que abarcan una variedad de contextos, poblaciones objetivo y enfoques. La mayoría de los estudios se basan en modelos matemáticos y demuestran que las medidas preventivas analizadas son costo-efectivas. Las intervenciones dirigidas a poblaciones de alto riesgo han mostrado los resultados más favorables. La generalizabilidad y transferibilidad de los hallazgos son limitadas debido a la heterogeneidad de las poblaciones, los escenarios y de los métodos utilizados. Además, los resultados dependen en gran medida de las hipótesis de modelización. Dado que las pruebas están distribuidas de forma desigual, se discuten las implicaciones para una futura investigación.

## Introduction

Sexually transmitted infections (STIs), and HIV in particular, are a significant public health issue. Effective prevention is critical to ending the HIV epidemic and combatting STIs. Evidence on the cost-effectiveness of prevention interventions and programmes is vital: In the context of limited resources, policy-makers need to balance the costs and benefits of preventive interventions [1].

Economic evaluations are important to decision-making. By quantifying and comparing the costs and outcomes of different interventions, they help to identify measures that offer the best value for money [2]. Evidence on cost-effectiveness is essential not only for priority setting [3], but also for the successful implementation of programmes [4]. However, the high number of economic evaluations (such as cost-effectiveness, cost-utility, or cost–benefit studies) makes it hard for decision-makers to maintain a broad perspective. A bibliometric analysis conducted in 2019 identified 372 economic evaluations in the field of HIV/AIDS alone, and highlighted various discrepancies in the existing evidence, as well as geographical and methodological heterogeneity [5].

Therefore, we aim to support decision-making by examining the ‘extent, range and nature’ of research activity [6, 7]. A scoping review is a useful and increasingly popular approach to map the evidence available in broad fields [8, 9]. The objective of the present scoping review is twofold: (1) to summarise the evidence on the cost-effectiveness of different interventions in the field of HIV/STI prevention to identify the most promising measures; and (2) to map the research field and identify potential shortcomings as well as research gaps.

## Methods

We performed this scoping review following the guidelines of the Preferred Reporting Items for Systematic Reviews and Meta-Analyses (PRISMA) Statement to systematically identify relevant studies (see research protocol, S1). An advisory group accompanied the process to ensure relevance for policy-making.

### Search Strategy and Inclusion Criteria

In May and June 2019, we electronically searched the Web of Science (WoS) and the Cost-Effectiveness Analysis (CEA) Registry. Tailored to the requirements of each database, the search strategy was based on the following three concepts: (1) HIV and/or STI; (2) preventive interventions; and (3) economic evaluation. For WoS, we used the following search algorithm:

(HIV OR HIV/AIDS OR AIDS OR HIV/STI OR STI OR HIV/STD OR STD) AND prevent* AND (cost* OR spend*) AND (effective* OR consequence* OR utilit* OR benefit* OR efficiency OR (economic AND evaluation))

For the CEA registry, only a basic search option was available. Therefore, we entered the different types of STIs separately and checked all entries for studies according to predefined inclusion criteria.

We formulated the inclusion criteria according to PICO (population, intervention, comparison, and outcome) guidelines. We included economic evaluations of preventive interventions or programmes targeting the general population, risk groups, or infected persons. We included any research designs of full economic evaluations—such as cost–benefit analysis (CBA), cost-effectiveness analysis (CEA), or cost-utility analysis (CUA)—if based on a comparison between different interventions or on/off comparisons. We did not predefine outcomes of HIV/STI prevention a priori and included measures such as averted infections, behavioural changes, and quality adjusted life years (QALYs). We only included studies focusing on Western, high-income democratic countries with concentrated epidemic settings in order to have a comparable context, not only with respect to the policy resources available, but also with regard to transmission patterns. Further, we only considered studies conducted after 1998 and up until 30 June 2019. We chose the 1998 starting point since lifetime financial and utility costs of HIV infections dramatically changed after the introduction of effective antiretroviral therapy (ART). Finally, we only considered English language articles published in peer-reviewed journals.

### Study Selection and Data Extraction

**Fig. 1:**
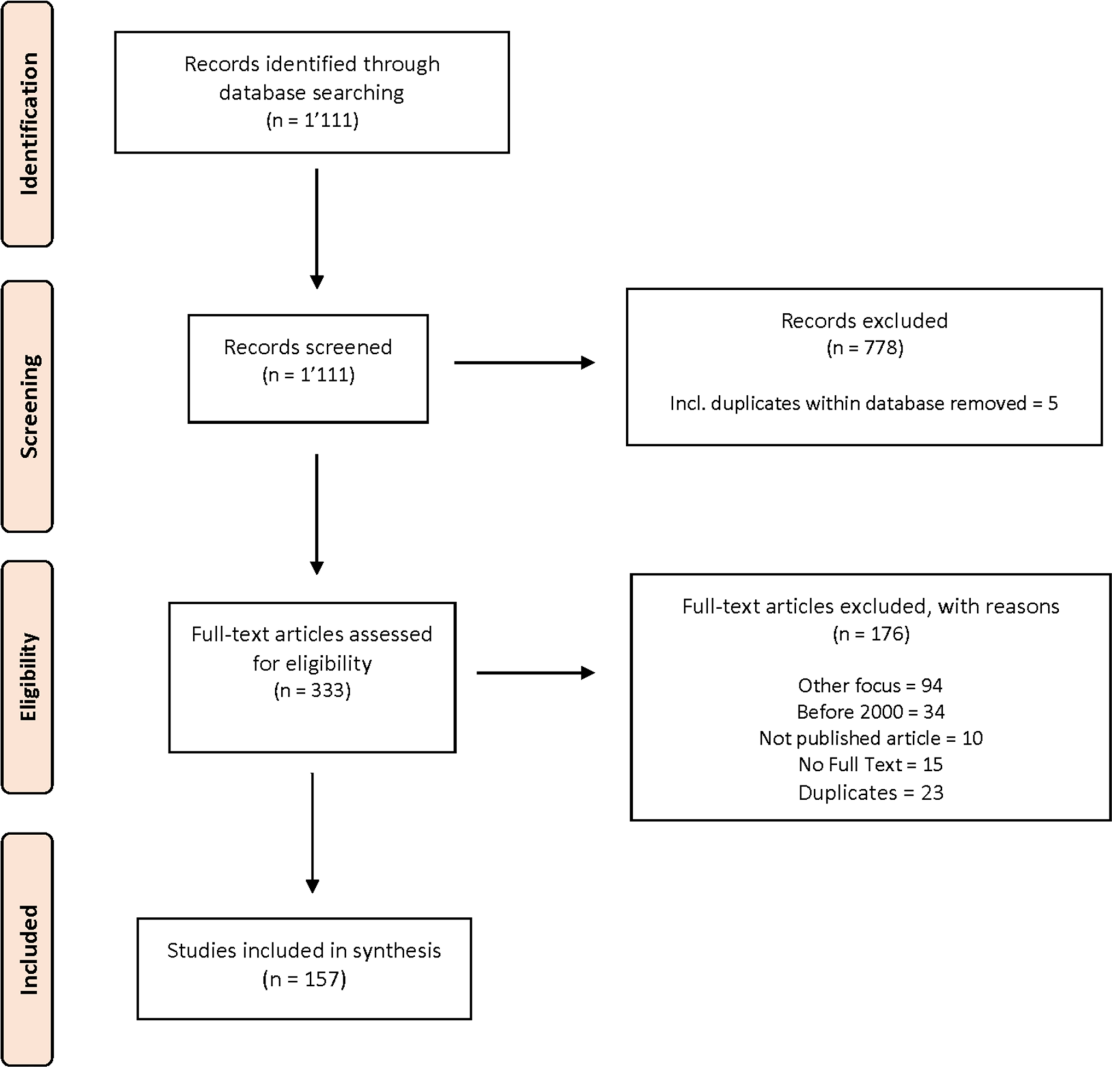
Selection of included studies based on the PRISMA flow diagram

We selected eligible publications using the multistep approach illustrated in Fig. 1. First, one author (PB) screened all titles and abstracts for eligibility. Then, full papers were obtained for those that appeared potentially relevant and were available. In the next step, the second author (KB) assessed the full text of each article and double-checked for inclusion according to the predefined criteria. Ambiguous cases were discussed between PB and KB, and the third author (DK) was consulted in cases of conflict or uncertainty.

The focus of the study was HIV/STI prevention but not treatment, and we only included studies related to treatment if explicitly researching treatment-based prevention. Furthermore, we excluded studies on the effectiveness of interventions that did not evaluate monetary aspects; nor did we consider economic evaluations containing only a cost description but not assessing the effects. Last, we excluded studies based on data collected mainly before the introduction of effective ART regimens, resulting in the exclusion of studies published earlier than 2000.

For all included publications, we abstracted the following data: formal criteria, focus of the study, target group, intervention, outcome, study design, and relevant details regarding cost effectiveness. Cost-effectiveness indicates a society’s willingness to pay for health gains and thus depends on the unique local aspects of the specific intervention being evaluated. Consequently, we did not use a common threshold to assess cost-effectiveness, but rather reported cost-effectiveness results based on the main findings of the included articles. Further, we refrained from assessing the quality of the methods applied, since the diversity in methodology, assumptions, and models used to estimate the outcomes across studies and interventions makes it difficult to establish quality. However, we reported whether sensitivity analysis was performed and whether limitations were addressed. In addition, we noted whether funding details were disclosed.

Data were abstracted by one author (KB) using a standardised form based on a codebook developed, tested, piloted and refined by all authors, together with the members of the advisory group (see S2). A second author (PB) independently checked the data for consistency and clarity. Uncertainties in coding were discussed among all three authors. Since coding was done mainly by one author (KB), the reliability of coding was assessed as intracoder reliability: After completion of coding, ten studies were selected randomly and coded again by the same coder (KB). For these ten studies, Krippendorf’s alpha for intracoder reliability was 0.882, which is clearly above the conventional minimal value of 0.61.

### Results

Our search in the two databases yielded a total of 1111 records from which we excluded 778 articles in the title and abstract screening process. Of the 333 remaining potentially relevant articles, we excluded 153 after full-text assessment. After removing 23 duplicates, we identified 157 relevant studies.

The subsequent review proceeded in two steps. First, we mapped the overall field regarding not only focus, type of preventive intervention, target population, and outcomes but also country region, year of publication, and other study characteristics. Second, we summarised the results of the studies for different intervention types separately, discussed the reported effects, and formed a narrative synthesis. (See S3 for an overview of all included studies.)

### Mapping the Field: Characteristics of the Included Studies

The number of economic evaluations of interventions in the field of HIV/STI prevention has increased since 2000, with an average of eight publications per year, peaking at 16 publications in 2018. Papers appeared in a plethora of journals. Outlets with ten or more studies included *AIDS*, *Sexually Transmitted Diseases*, *AIDS & Behaviour* and *PLOS ONE*. The prevalence of studies conducted in the North American context was striking (62%), with most of them originating in the US (56%). Thirteen percent of the studies had an international focus covering more than one country. Fewer economic evaluation studies on HIV/STI prevention have been conducted in Western European countries (20%), whereby the UK (8%) and the Netherlands (7%) head the list.

Most studies entailed a form of mathematical modelling (82%) including decision analytic models, threshold analyses, Markov and deterministic models, static and dynamic models, and individual- and population-based models. Systematic reviews were the second most frequent design (15%). When deciding which types of costs and benefits to include in an economic evaluation, the most common were a societal perspective (33%) and a health care system perspective (27%). Most studies employed an annual discount rate of 3%.

The main focus of the studies was HIV, and only 12 studies each were published on chlamydia and hepatitis B/C, whereby studies on gonorrhoea (3), syphilis (2), or STIs in general (6) were less common.

**Fig. 2:**
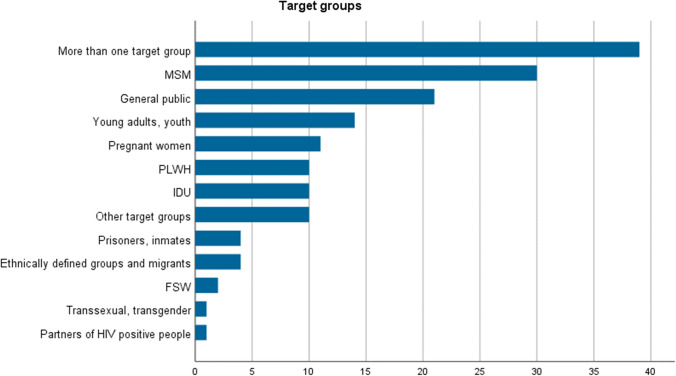
Number of studies on different target groups

With respect to target groups (Fig. 2), the largest share of studies analysed interventions for more than one target group (25%), followed by 30 papers focusing on men who have sex with men (MSM). The general public, youth, and pregnant women accounted for one-third of the studies. Many fewer studies, however, dealt with other risk groups such as injecting drug users (IDUs), prisoners, female sex workers (FSWs), or people living with HIV (PLWH) and their partners. Other targeted groups included mentally ill persons or older individuals.

**Fig. 3:**
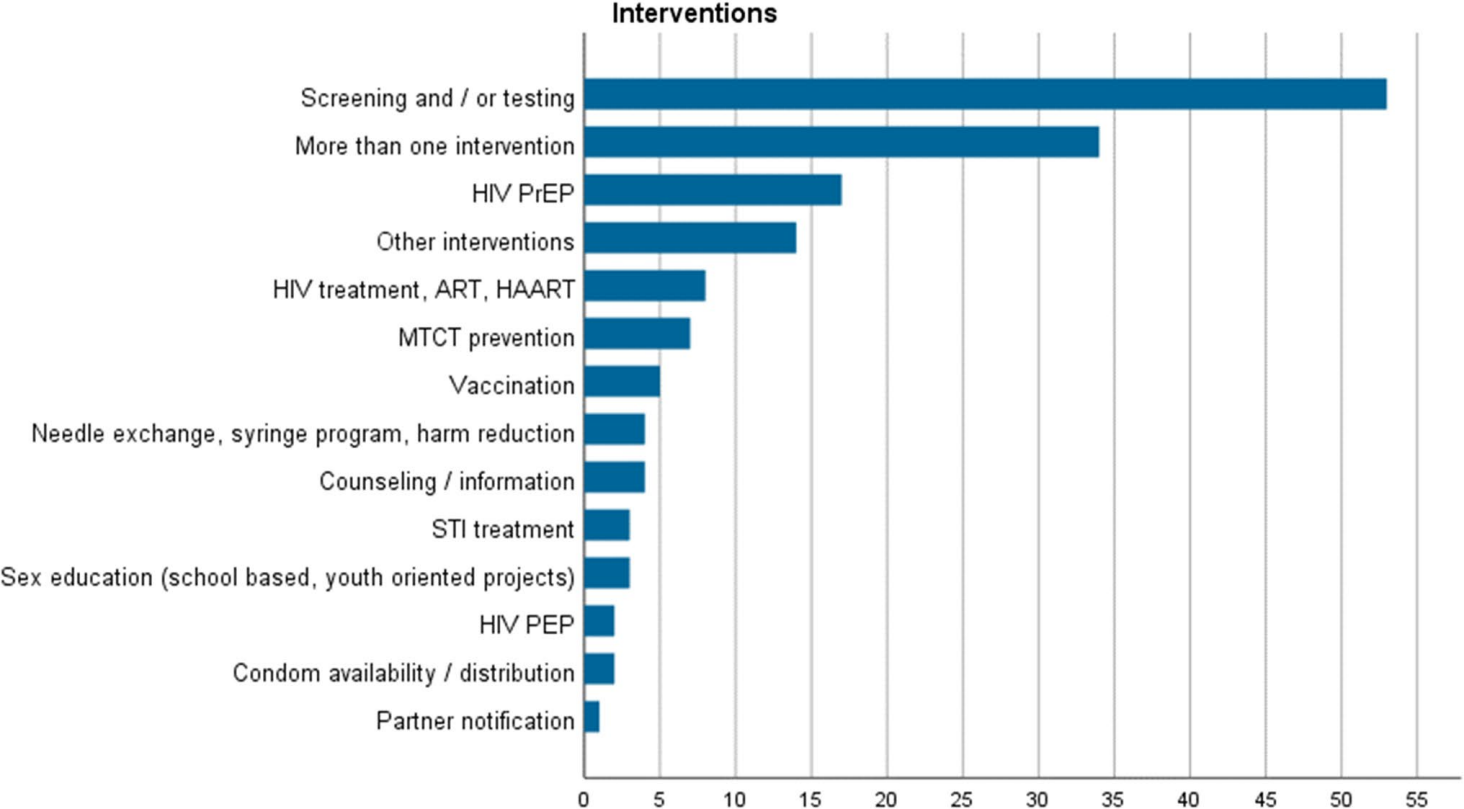
Number of studies on different intervention types

Regarding interventions (Fig. 3), the largest share of studies focused on screening and/or testing as a preventive measure, and 10% of the included studies dealt with pre-exposure prophylaxis (PrEP), i.e. medication taken to reduce the risk of HIV transmission via sex or injection drug use. A total of 34 studies analysed more than one intervention. In conclusion, there is substantially more scientific evidence on biomedical interventions than on structural approaches or measures aimed at behavioural changes. One possible explanation is that the effects of biomedical interventions are more straightforward to study but also include more recent approaches and developments.

One of our selection criteria was that a study estimated the cost per unit of effect for each intervention under scrutiny. The most commonly used outcome variable was the number of infections prevented (27%), but measures such as the number of QALYs gained (12%) or the averted costs of (future) treatment (7%) were also used. However, a majority of studies included more than one indicator for outcome measurement (42%). Here, again, methodological considerations might be decisive for measurement choice. Indeed, only 2% of the studies included in this review focused on a decrease in risky behaviour as a primary outcome variable, which is difficult to measure.

Looking at the chief conclusion of the included studies, only 4% reported that the intervention under examination was not cost-effective, whereas 6% did not explicitly give any assessment. Almost one-third (29%) concluded that the evaluated interventions were cost-effective, and 6% found them to have been cost-saving. Forty-three percent concluded that the interventions assessed might be considered cost-effective and/or cost-saving under certain conditions (such as reduced drug prices) or reported more than one outcome (12%). This finding comes as no surprise since a majority of the studies were based on mathematical modelling and therefore compared different scenarios. However, it is important to note that the reviewed papers used different definitions and thresholds of cost-effectiveness.

The majority of the studies contained an explicit discussion of potential biases and limitations and included sensitivity analyses. The source of funding was not disclosed in 45% of the studies (it was disclosed in 21%), and 34% of the studies explicitly discussed the (non)involvement of the funding source.

### Findings on Different Prevention Interventions: Narrative Synthesis

In the second step, we categorised the 157 studies into three main areas of HIV/STI prevention: (1) structural approaches; (2) interventions aiming at behavioural changes; and (3) biomedical interventions. Within these three categories, we made a further distinction between different subcategories in a narrative synthesis. We discuss studies covering several categories or subcategories of interventions according to their central emphasis. Additionally, we separately list measures to prevent mother-to-child transmission (MTCT). Finally, we assess studies comprising comparisons or combinations of different measures in the last section.

#### Structural Approaches

Structural interventions seek to address contextual aspects that facilitate or hinder the prevention of HIV or STI infections, such as socio-political, economic, or legal factors [10]. This approach therefore aims to prevent transmission by altering the environment, rather than by targeting risky behaviour [10]. Among the 157 studies included in this review, fifteen concentrated on structural approaches: three on broad national policies, four on needle exchange programmes, three on condom distribution, four on linkage and retention programmes for PWLH, and one compared different structural approaches. They show that these interventions not only reduce infection numbers, but also that they are cost-effective.

##### Broad Policy Initiatives and National Programmes

Two studies evaluated broad national programmes in the US and found generally positive outcomes. The national gonorrhoea control programme for the general population was found to be cost-saving if the benefits of gonorrhoea prevention and other benefits were considered [11]. The authors of the study estimated that 32 million infections with gonorrhoea were averted over a 33-year period due to prevention measures including disease surveillance, clinical services, the provision of condoms, counselling and partner services, and the implementation of behavioural change interventions. Additionally, a study analysing a US capacity-building programme concluded that community-based organisations can meet cost-effectiveness thresholds given the lifetime treatment costs of HIV [12]. Last, in an economic evaluation of community-based HIV prevention programmes in Ontario, Canada—which included education, campaigns, and social support—programmes were found not only to reduce the number of HIV infections, but also to reduce health care costs [13].

##### Harm Reduction and Needle Exchange

Four studies focused on needle-syringe exchange programmes (NSPs). A review established that NSPs reduce HIV transmission in intravenous drug users not only in an effective and safe way, but also in a cost-effective manner [14]. In a similar vein, an Australian study found that NSPs are likely to avert a substantial number of not only HIV, but also hepatitis C virus (HCV) infections among IDUs, and are thus cost-effective in the short term and even cost-saving when future health outcomes and costs are taken into account [15]. Concerning HCV, NSPs in the US were found to have little impact on HCV incidence and prevalence [16]; however, in a UK study, this intervention was considered highly cost-effective (<GBP  20,000 per QALY saved) [17]. HCV transmission among IDUs also depends on how comprehensive a harm reduction model is and whether it is coupled with referral of IDUs to treatment [16].

##### Condom Distribution

Condom distribution programmes are a structural-level intervention aiming to increase the availability of condoms and their use. Three studies focusing on condom distribution programmes suggested that such programmes are cost-effective. If targeted at young people [18] or if conducted at a community level [19], such programmes might even be cost-saving. The same applies to a condom distribution programme targeted at women that was combined with education [20].

##### Linkage and Retention Programmes

The four studies assessing linkage to, re-engagement with, and retention in care programmes for people living with HIV were all conducted in the US and based on mathematical modelling: access to care [21], early linkage [22], and linkage of HIV-infected jail releases to community HIV care [23] were found to be cost-effective. HIV linkage and retention programmes are thus an efficient investment from a societal and payer perspective since the thresholds appear achievable [24].

##### Comparison of Structural Interventions

One study comparing different structural interventions aiming to prevent HIV in women in the southern US found all of these to be cost-effective compared with average lifetime HIV treatment costs [25]. Mass media campaigns, condom availability, and alcohol taxes prevented the largest numbers of HIV infections and were found to be cost-effective at less than 10,000 USD per infection prevented.

#### Behavioural Interventions

Behavioural strategies are defined as interventions seeking to motivate behavioural change. Most of them aim to reduce the risky behaviour of infected individuals or of those at risk of HIV/STI transmission. The results of the 18 studies in this category imply that behavioural interventions are cost-effective and potentially cost-saving; they reduce the risk of HIV transmission, and the savings resulting from averted HIV infections far exceed the costs of the interventions.

##### Counselling

The fifteen economic evaluations on counselling focused on interventions targeting PLWH (2), MSM (3), IDUs (4), FSWs (1), prisoners (1), mentally ill persons (2), and others (2). They show that counselling can be cost-effective, especially if targeted at high-risk groups.

A study reported that clinical provider-led counselling for *PLWH* was cost-effective in reducing sexual HIV transmission risk compared to other types (by specialists or mixed) [26]. Further, holistic and individually based behavioural interventions for HIV-positive MSM with uncontrolled virus could potentially be cost-saving because of improvements regarding adherence as well as psychosocial concerns [27].

Another counselling intervention to improve adherence to ART among *MSM* was found to be cost-effective; even though counselling produced only modest benefits in terms of prevention, it provided significant benefits for individual patients at an affordable cost [28]. A systematic review suggested that individual-level, group-level, and community-level HIV behavioural interventions are cost-effective in reducing HIV risk behaviours among MSM by reducing unprotected anal intercourse and increasing condom use [29]. Similarly, interventions targeting young gay men were found to be cost-effective, since the cost for averting one HIV infection is by far inferior to the lifetime medical costs of HIV disease, also when compared with other prevention strategies [30].

Threshold analyses of interventions with sexual and injection risk-reduction components targeted at *IDUs* and their sexual partners in the US found these to be cost-effective or even cost-saving since they have the potential to save substantial economic resources by averting HIV-related expenses [31]. For women using drugs, a standard intervention (pre- and post-test counselling) combined with a ‘well-women exam’ (an additional breast and routine pelvic examination with cervical cytological testing) is cost-effective for preventing hepatitis C and gonorrhoea [32]. One study found that behavioural interventions were effective and cost-effective at reducing HIV incidence among both HIV-positive and other IDUs [33]. Similarly, a project conducted in four US cities led to modest changes in sexual risk behaviours of HIV-positive IDUs [34].

A brief behavioural intervention was found to reduce the incidence of HIV and STIs among *FSWs* in a cost-effective manner [35]. If ART is included in the model, an annual intervention strategy even results in net savings.

Other researchers have examined what thresholds of a single- and multi-session behavioural intervention targeting young *imprisoned* men (who will be released from jail) need to be achieved to be cost-effective; they found that costs are comparable with other HIV interventions and that the thresholds should be achievable [36].

Economic evaluations of behavioural interventions targeted at *adults with (severe) mental illness* contained mixed conclusions but highlighted gender differences, and thus the importance, of focusing on gender-specific issues when planning HIV prevention interventions. For instance, a small group intervention aimed at risk reduction in the US did not result in significant condom use uptake by men, but was slightly cost-effective if directed toward high-risk, sexually active women [37]. Similarly, while advocacy training was the most cost-effective way to reduce risky behaviour in men, only single-session one-on-one interventions were found to be a cost-saving intervention in women [38].

Last, other studies included a community-level intervention targeting women in low-income housing developments in different US cities, showing moderate cost-effectiveness in comparison with other HIV prevention programmes for at-risk women [39]. Finally, interventions were found to be usually more cost-effective if they were initiated early on in the epidemic [40].

##### School-Based Interventions

An economic evaluation of school-based interventions to prevent sexual risk behaviour concluded that such interventions can be cost-effective, but that local factors such as programme costs, prevalence and incidence rates, as well as medical costs, may qualify this conclusion [41]. School-based behavioural interventions are likely to bring improvements in knowledge and increased self-efficacy depending on the quality of intervention providers, their enthusiasm and expertise, and a supportive school culture that recognises the individual sexual health needs of young people [42]. However, evidence suggests that the reduction of sexual risk behaviour and infection rates is only moderate [42], and that the effect of the intervention on sexual behavioural outcomes is uncertain [43].

#### Biomedical Interventions

The majority of the studies included in this review (101 of 157) focused on biomedical interventions to prevent the transmission of HIV or other STIs. These cover testing and screening for the general population and at-risk groups (56), treatment as prevention (9), a so-called ‘test and treat’ combination (5), vaccination (6), and the use of antiretroviral drugs by HIV-uninfected persons to reduce the risk of HIV transmission pre- and post-exposure (25).

##### Testing and Screening

Early identification of HIV infections and other STIs is critical not only because of better treatment options, but also to reduce the transmission from infected individuals to others. However, a significant number of people are unaware, for example, of their HIV status [44]. Given the well-established clinical and public health benefits of early detection, it comes as no surprise that with 56 studies out of 157 included in this scoping review, the largest number were concerned with testing and screening strategies. Of those, three were systematic reviews; the others were based on mathematical modelling. The most common outcome measure was the estimated cost per QALY gained, and some studies reported the costs per averted or identified infection. The subsequent synthesis involves subcategories according to the target population of the assessed interventions: the general population (16) and young adults (9), populations at higher risk, including partners of PLWH (3), MSM (8) and other at-risk groups (10). The last subcategory encompasses studies comparing different testing strategies or target groups (10). In sum, the studies indicate that expanded HIV/STI screening leads to earlier access to treatment, which then results in a decrease in morbidity and mortality, as well as a reduction in the transmission of HIV/STIs. Less is known, however, about whether reductions in risk behaviour occur among those tested. The debate of whom to test and how frequently remains controversial in the studies reviewed here. Nevertheless, they offer concurring evidence that repeated screenings of high-risk populations provide good value for money, and that one-time screenings of populations with a low prevalence of STIs might be a sound public health investment. Testing is highly effective, especially if diagnosis is followed by prompt linkage to medical care.

*STI screening in the general population:* Sixteen studies assessed testing and screening strategies of STIs for the general population. Twelve focused on HIV, three on chlamydia, and one on HCV. They tended to imply that universal screening strategies in the general population, although costly, can be cost-effective, especially when they involve community-based one-time routine testing or programmes in emergency departments (ED). Focusing on HIV screening measures in a European context, a Dutch study showed that universal HIV screening of patients attending an STI clinic in Amsterdam had an acceptable cost-effectiveness estimate [45]. The cost-effectiveness of routine HIV screening in Portugal compared to the current approach (targeted and on-demand screening) was evaluated in another study [46], which concluded that one-time HIV screening in the general population would be a cost-effective measure and repeated screening in higher-risk regions and subpopulations a justified option. A UK study assessed the trade-off between benefits of an early HIV diagnosis—resulting in a decrease in morbidity, mortality, further transmission, and costs—and increased treatment costs [47]. The findings suggest that HIV screening in primary care settings is cost-effective in the UK in the medium term. In the US context, several studies focused on the impact of preventive interventions recommended in Centers for Disease Control (CDC) guidelines, including routine HIV counselling, testing, and referral for patients with an HIV prevalence of ≥ 1% [48]. The authors found that routine HIV screening programmes in health care settings would most likely also remain cost-effective at a lower HIV prevalence [48]. Other studies showed that one-time screening for the general population [49] and routine, voluntary HIV screening for all is cost-effective [50], with incremental cost-effectiveness ratios of USD 30,800/QALY (one-time screening), USD 32,300/QALY (screening every 5 years), and USD 55,500/QALY (screening every 3 years) [51]. Another study found that an expanded HIV testing policy would have a large impact on government budgets and concluded that providing care for newly identified cases is likewise crucial [52]. An opt-out HIV testing—i.e. testing without the need for risk assessment and counselling—in all health care encounters for persons aged 13 to 64 was assessed in another study [53], which reported that even though potentially thousands of new infections would be detected, targeted counselling and testing still led to better performance. In another publication, this opt-out rapid HIV screening strategy was compared with physician-induced diagnostic rapid HIV testing in an urban ED, showing the former to be more costly but at the same time identifying more HIV infections [54]. Other studies focusing on the specific context of EDs found that targeted HIV screening can be a cost-saving option since it increases quality-adjusted life expectancy [55]. A review of economic evaluations on HIV screening in emergency departments emphasised that although many studies report cost outcomes, it is difficult to compare results [56]. However, many studies in the abovementioned review indicated that HIV screening in ED-based programmes is a cost-effective strategy. With regard to other STIs, a study in France compared HCV universal screening to the current strategy of screening only individuals at high risk of infection and found the latter to be cost-effective from a societal perspective [57]. Studies focusing on chlamydia prevention assessed a range of interventions. One study suggested that programmes targeting venues that have access to men at high risk could be cost-effective since men often transmit chlamydia [58]. Another study showed that a community-based intervention in Sweden called ‘Chlamydia Monday’—consisting of the provision of information and increased availability of testing, treatment and contact tracing—was cost-effective [59]. The last study focused on rectal chlamydia trachomatis screening as an add-on to female urogenital screening in STI clinics in Canada, and highlighted that selected and universal rectal screening are cost-effective compared to universal urogenital-only screening strategies [60].

*Screening of young adults*: Nine studies focused on screening young adults, mainly concerning chlamydia but also other STIs such as gonorrhoea, hepatitis B virus (HBV), HCV, and syphilis. These studies focused on a variety of settings, which makes general conclusions difficult. One study indicated that screening young women for gonorrhoea in US urban EDs is cost-effective and, because of considerable prevalence in this patient group, could prevent substantial reproductive morbidity [61]. Another publication focused on a school-based STI screening programme and showed that a chlamydia screening programme was cost-effective and could even be cost-saving in some settings [62]. Comparing different screening approaches for chlamydia trachomatis, one study found that the most cost-effective strategies were annual screening in all young women and targeted semi-annual screening for those with a history of infection [63]. In a similar vein, an economic evaluation of the National Chlamydia Screening Programme in England—which includes annual testing offered to men and women aged under 25—found that under accepted thresholds, the current programme is the most cost-effective strategy compared to alternative approaches [64]. The cost-effectiveness of different screening strategies for Dutch young adults was analysed in another study, which showed that screening every two years would be optimal [65]. In contrast, repeated screening of chlamydia in the Netherlands was found to be cost-effective only with a willingness to pay threshold of > EUR 50,000 per QALY gained [66]. Similarly, a modelling study indicated that an opportunistic chlamydia screening programme was unlikely to be cost-effective in Ireland and too expensive to implement [67]. Last, a study in Scotland, where chlamydia is the most common STI, revealed that current chlamydia testing strategies, focusing on individuals with symptoms or at high risk, are not cost-effective [68]. A more restrictive testing strategy was assessed in the Netherlands, where syphilis and HIV tests for younger, heterosexual clients were limited in 2015 due to rising costs of care [69]. This policy resulted in only a slight decrease in detected infections and in savings of EUR 435,000 per QALY lost. Further, the authors found it would be cost-effective to offer tests for syphilis and HIV to first- and second-generation immigrants and to conduct HIV testing in case of a positive chlamydia or gonorrhoea diagnosis.

*Testing in populations at higher risk:* A total of 21 studies assessed screenings for individuals with higher risks of STI infection. Their results vary across settings and target groups. However, the evidence suggests that testing strategies targeted at specific populations at high risk of HIV/STI infection are cost-effective. Three studies examined *partner notification*, i.e. preventing HIV transmission by notifying sexual partners of an HIV-positive person. In an analysis of partner notification programmes for Dutch MSM, researchers found that partner notification strategies can be expected to prevent new infections in an increasingly cost-effective manner over time [70]. More particularly, this study considered online partner notification to be a useful tool to get individuals at high risk to test for HIV. In a US study, partner counselling and referral services were examined, concluding that programme costs of point-of-care rapid HIV testing varied substantially by location [71]. Another telephone-based partner notification approach of prevalent STIs would cost USD 4499 per DALY; thus, the implementation of selective screening with partner tracing is seen as a cost-effective intervention [72]. Eight studies focused on HIV/STI screening targeted at *MSM*, five in the US, two in the Netherlands, and one in the UK. In one of the Dutch studies, the authors assessed annual anorectal chlamydia screening among MSM in care at HIV treatment centres, and found this added screening to be cost-saving if only a limited proportion of men were not routinely screened [73]. In another Dutch study, testing strategies for the detection of anogenital gonorrhoea among MSM were analysed, and the authors found that a gram-stained smear for symptomatic MSM was a cost-effective option [74]. The UK study evaluated a ‘recall for rescreening’ strategy and showed that this approach of rescreening MSM diagnosed with an STI leads to high screening rates and detections of new infections [75]. In an urban US context, rectal screening of MSM for chlamydia and gonorrhoea was considered potentially cost-effective if targeted at MSM at risk [76]. Moreover, syphilis screening among MSM is considered a cost-saving option to prevent HIV infections [77]. Three other US studies focused on HIV screening of MSM. The first recommended HIV testing in MSM with an influenza-like illness in addition to encouraging annual antibody screening [78]. Furthermore, one study reported that community-based acute HIV testing compared to HIV antibody testing alone is cost-effective in preventing new infections among at-risk MSM [79]. Last, the authors highlighted that HIV testing should reach a cost-saving threshold if the programmes can reach undiagnosed PLWH; hence, programme cost utility could be maximised if strategies target MSM who are most likely to have undiagnosed HIV [80]. A study on screening strategies targeting *transgender persons* assessed the costs and effectiveness of rapid HIV testing services in New York City and San Francisco [81]. The authors found that even though the programmes had relatively high fixed costs and required a substantial investment, testing services provided to transgender individuals helped to identify a high proportion of new HIV diagnoses among the persons tested. However, there were differences in the average costs per infection detected between the two sites (USD 3563/USD 8284), which was explained in the proportion of previously undiagnosed HIV infection among those tested. Regarding soon-to-be-released *prisoners*, offering HIV counselling and testing is seen as a cost-saving prevention measure [82]. An Australian study on regular and mandatory screenings of *sex workers* found this testing strategy to not be cost-effective since the incidence and prevalence of STIs in this population are very low because of almost universal condom use [83]. Thus, the authors recommended that screening frequencies for sex workers be based on local STI epidemiology. Two studies on rapid testing in *substance abuse* treatment programmes showed that both on-site rapid HIV testing [84] and on-site rapid HCV testing [85] increase life expectancy in a cost-effective manner (< USD 100,000/QALY gained). Last, three studies focused on testing strategies in *migrants*, especially those born in countries with high STI/HIV prevalence. A systematic overview of the cost-effectiveness of HIV testing strategies in migrant populations in the European Economic Area concluded that community-based rapid testing programmes potentially improve access to and uptake of testing [86]. HBV/HCV screening targeted at foreign-born migrants from low endemic countries was found to be a cost-effective measure to enhance early identification and treatment (< EUR 10,000/QALY gained) [87]. A UK study on enhanced HBV screening among migrant populations concluded that a ‘one-time opt out case-finding approach’ in primary care settings is very likely to be cost-effective (approximately GBP 20,000/QALY gained) [88]. A study on notifications of a new HIV diagnosis through a social network strategy among *minority populations* found costs to vary strongly across sites [89]. Finally, an assessment of increased spending for HIV counselling and rapid testing in *high-risk communities* in the US indicated favourable effects on public health benefits and cost savings to society [90].

*Comparison of testing strategies and target groups:* Ten studies compared different testing strategies. Semi-annual testing via fourth-generation immunoassay was found to prevent a greater number of infections and to be more economically efficient than annual nucleic acid amplification testing strategies of MSM and IDUs [91]. Comparing the cost-effectiveness of HIV screening in different settings, one study reported that testing MSM in STI clinics and screenings in EDs was more cost-effective than diagnosis based on clinical manifestations, since PLWH are identified at less-advanced stages of HIV infection [92]. HIV testing for high-risk seronegative individuals (young and old MSM, IDUs) is a costly intervention, but might be cost-effective if applied to a large high-risk population [93]. Another study comparing targeted and routine testing strategies found that the former resulted in higher positivity rates, but that routine testing led to a higher overall number of infections detected, concluding that a combined testing strategy is preferable [94]. The cost of identifying a new HIV diagnosis is, however, substantially higher in an outreach setting than in a clinical setting [95]. Evaluating three testing strategies, such as rapid HIV testing in clinical settings, community-based organisation (CBO) settings, and the Partner Notification Services programme, another study found that expanded testing programmes are cost-saving overall [96]. Regarding the frequency of testing, the CDC guidelines (a one-time test for low-risk individuals and annual testing for those at high risk) were considered too conservative by one study, whose authors advocated for more frequent testing as a cost-effective measure for all risk groups [97]. An assessment of the frequency of screening for different at-risk groups found quarterly HIV testing in MSM to be more cost-effective than annual testing, but more frequent than annual testing for IDUs to be not cost-effective [98]. In the UK, annual HIV testing of at-risk populations was found to be very cost-effective, and the identification of undiagnosed PLWH could be improved through additional one-time testing of all other adults [99]. A comparison of HCV screening strategies for different target groups in the Netherlands [100] revealed that a strategy aimed at the general population was not cost-effective, but a strategy accompanied by additional primary care support, as well as strategies targeting hard drug users, were cost-effective.

##### Treatment as Prevention (TasP)

The treatment as prevention (TasP) strategy has gained importance and is seen as a win–win strategy not only due to individual health but also public health benefits [101]. Studies in this category not only support the potential of TasP to reduce the number of new HIV infections but also the economic benefits, despite its high costs.

Nine studies had an international focus and discussed the importance of TasP for the global goal of ending AIDS, whereby a further scale-up of treatment could produce greater economic benefits despite the high costs [102]. However, although TasP might be cost-effective, determining factors vary across settings [103], and its implementation strongly depends on governments being able to afford the costs [104], stressing the potential of generic drugs. Cost implications as well as effectiveness data from different settings were reviewed in a study that found that TasP strategies rely on complementary interventions to reduce new HIV infections [105]. One of these is providing access to care and ART to PLWH who do not have health insurance, the main goal of the US AIDS Drug Assistance Program, which was found to be cost-effective [106]. Similarly, studies in Canada indicated that an ART expansion scenario was associated with net benefits [107] and that ART scale-up has decreased HIV-related morbidity, mortality, and transmission and thus is cost-saving from a societal perspective [108].

Last, one study was concerned with HCV infection therapy for HIV-infected MSM in the Netherlands and concluded that treatment with direct-acting antivirals is crucial to preventing new infections since cured individuals cannot transmit HCV [109].

##### Combination: Test and Treat

Five studies focused on a combination of testing and treatment strategies. A US study on HCB found that an inclusive approach encompassing screening and treating or vaccinating was cost-effective for different high-risk, high-prevalence populations [110]. Focusing on HIV-positive women, who have a high incidence of Trichomonas infections, screening and treatment for the purpose of decreasing HIV transmission to male partners was found to be cost-saving [111].

From a societal angle, an expansion of HIV screening and treatment is cost-effective: A combination of those approaches in the US could prevent 17% of infections and cost USD 21,580/QALY gained, even though changes in risk behaviour are also necessary to reduce the HIV epidemic significantly [112]. Likewise, another study found HIV testing and interventions used to foster treatment initiation to be cost-effective in contrast to ART retention initiatives [113]. Preventive measures target the various stages of the HIV care sequence; however, there are still substantial gaps in knowledge regarding how to maximise the value of health spending [114].

##### Vaccination

The development of an effective vaccine is often seen as the holy grail of preventing HIV/STIs [115]. Six studies included in this scoping review assessed the effects of hypothetical vaccinations. They indicated possible benefits even of modestly effective vaccines, especially when vaccination is targeted at high-risk groups.

Looking at an adult population in STI clinics, one study found that substituting hepatitis A/B for hepatitis B vaccines would reduce morbidity and mortality in a cost-effective manner [116]. With regard to HCV, even a moderately effective vaccine is seen as cost-saving in high-risk groups and economically attractive in lower-risk cohorts [117]. An effective chlamydia vaccine for young women would also be cost-effective in the US [118].

With regard to HIV, one study suggested that HIV chemoprophylaxis among high-risk MSM could prevent a significant number of HIV infections and be cost-effective [119]. Similarly, some studies assumed that partially effective vaccines for at-risk groups (MSM and IDUs) would result in net cost savings [120], and vaccination strategies could have a great impact on reducing new infections among MSM in Australia [115].

##### Pre- and Post-exposure Prophylaxis (PrEP and PEP)

*Pre-exposure prophylaxis* (*PrEP*) refers to the use of antiretroviral medications in HIV-uninfected persons prior to HIV risk exposure. Since clinical trials, such as the PROUD in the UK [121] or the French/Canadian IPERGAY [122] studies, have shown a high efficacy of this preventive intervention, it is considered to have great potential to reduce population-level transmission. However, the high costs of PrEP have until recently been seen as the main barrier to its use. Hence, economic evaluations are crucial to determine whether, in what settings, and for which populations this rather new prevention strategy is a worthy investment.

Among the studies included in this review, 23 focused on the cost-effectiveness of PrEP. Eleven studies were conducted in the US, some had an internationally comparative focus or were European (one each in the Netherlands, the UK, France, and Germany), two were carried out in Canada [123, 124], and one in Australia [125]. With 14, the majority of the PrEP studies focused on MSM, and some included the general population or specific target groups such as IDUs [126, 127] or serodiscordant heterosexual couples seeking to conceive [124, 128]. Most of the studies used some form of mathematical modelling, and four were reviews.

The studies generally acknowledged the high potential of PrEP for HIV prevention [129], especially in younger or high-risk populations [130]. Earlier studies were more sceptical from an economic angle due to the high costs and because they assumed a lower efficacy of PrEP [131]; recent studies, however, have found that PrEP can be cost-effective [123]. Some authors have even suggested that under certain conditions, most notably when drug prices are reduced, PrEP is cost-saving [132–134].

Offering PrEP to specific subgroups and for periods when a person is at particularly high risk, such as an event-based PrEP programme [135], helps to increase cost-effectiveness. Another study predicted that costs could be reduced the most if any of the non-daily regimens (PrEP on demand) were implemented [136]. However, the identification and successful targeting of most at-risk groups is not always straightforward. One study found that targeting HIV-negative MSM in a discordant regular partnership is the most cost-effective intervention, even though this highly targeted strategy would not have a large population-level impact [125]. In contrast, for serodiscordant heterosexual couples seeking to conceive, condomless sex restricted to time of ovulation is the most cost-effective strategy [124], but PrEP is only cost-effective if HIV suppression is low [128].

Findings of economic evaluations on PrEP depend heavily on underlying assumptions about HIV transmission (sexual behaviour, adherence), as well as about costs of drugs and other contextual factors; this explains the varied results on cost-effectiveness [137]. The maximum benefit from PrEP results from its introduction in combination with other HIV prevention programmes, such as screening and immediate treatment of diagnosed infections [138], except for vaccines that, once available, would be more cost-effective than PrEP [139]. However, more research on the optimal combination of test/PrEP/treat is needed since high PrEP coverage with early ART is expected to provide the greatest benefit, but is also the most expensive strategy [140]. Additionally, some caution is necessary because the use of PrEP could potentially lead to decreased condom use, which could lead to an increase in infections with other STIs. On the other hand, PrEP programmes are seen as a good strategy to better screen and treat STIs. For instance, a French study found additional benefits associated with the introduction of PrEP, such as the treatment of other diseases and a reduction in secondary infections [141]. In sum, even though the cost-effectiveness results vary, it appears that PrEP can be a cost-effective addition to other HIV prevention programmes since it has the potential to prevent a substantial number of HIV infections and thus to mitigate the HIV epidemic [142]. Existing evidence suggests that the enormous upfront costs in providing PrEP should result in substantial economic benefits in the long term [143]. However, high current drug costs limit cost-effectiveness. Further, access to PrEP for the general population of at-risk groups would be very expensive. Hence, most studies propose prioritisation based on the self-reported risk behaviour [144] of those at highest risk of HIV exposure (between 10 and 30% coverage). Many reviewed studies performed sensitivity analyses and discussed limitations. For instance, data on changes in sexual risk behaviour, as well as adherence among people taking PrEP, would need to be taken into account to accurately assess the cost-effectiveness of PrEP [125]. Likewise, more information on sexual activity or drug resistance would be important. In particular, the lack of evidence on behavioural responses to PrEP [138], as well as the effect of its introduction on alternative prevention approaches [145], warrants further investigation.

Only two studies focused on *post-exposure prophylaxis* (*PEP*), i.e. the use of antiretroviral medications in HIV-uninfected persons after high-risk HIV exposure events. The two studies found that PEP was cost-saving for men who reported receptive anal intercourse (RAI), especially with an HIV-infected partner [146], and possibly cost-effective for injection-drug exposure and women reporting RAI [147].

#### Mother-to-Child Transmission (MTCT)

MTCT represents the major route of HIV infection for children. Twelve of the 157 included studies fell under this category. They show that universal screening of pregnant women for HIV and other STIs, such as chlamydia and syphilis, is a cost-effective measure to prevent MTCT. Both antenatal HIV screening and later rescreening are described as cost-effective or even cost-saving [148]. Studies suggest maximising screening coverage [149], even in cases of an extremely low HIV prevalence of 0.01% [150]. However, in certain settings where universal screening is not feasible, targeted rescreening of women with a history of high risks could be a valuable option. Moreover, prevention of MTCT is cost-effective if combined with drug treatment [32] or for women without prior prenatal care [151]. For a high-risk population of incarcerated pregnant women, routine prenatal screening is expected to significantly reduce MTCT [152]. Additionally, programmes that optimise adherence to treatment during pregnancy have the potential to diminish MTCT in a highly cost-effective manner [153]. Last, two studies reported that an elective caesarean section for HIV-positive women is considered a cost-effective intervention to prevent MTCT [154, 155], and for women with detectable HIV during pregnancy, it is associated with increased quality-adjusted life expectancy and lower costs compared with vaginal delivery. However, more recent evidence implies that for pregnant women with undetectable viral load under ART, vaginal delivery can be safe if breastfeeding is avoided [156].

Apart from HIV, MTCT involves other STIs. In an Australian study, researchers found that because of the increasing prevalence, screening of all young pregnant women for chlamydia was likely to be cost-effective compared with no screening or selective screening [157]. Further, two US studies focused on testing and screening pregnant women for STIs. The first determined, with the help of a decision analysis, the costs and benefits of screening young pregnant women for chlamydia trachomatis infections [158]. Considering a high prevalence (> 17%), prenatal screening is cost-saving, and with the current prevalence rates (7%), prenatal screening results in higher spending but also in a significant reduction in the morbidity of woman-infant pairs. In another publication, screening for syphilis was studied, showing more favourable results regarding cost-effectiveness as well as maternal and neonatal outcomes for repeated (versus single) screening strategies [159].

#### Comparisons and Combinations of Different Interventions

Even though economic evaluations on specific measures are valuable for policy-makers, a comparison of interventions or the effects of combinations of different measures can help to maximise the impact of their HIV prevention resources. Thus, comparing estimates of the cost-effectiveness of HIV interventions provides important insights regarding prioritisation. However, out of the eleven studies in this category, there were only two economic models and two reviews, which undertook a broad comparison among different prevention types. Seven studies compared interventions by focusing on specific populations.

##### Broad Comparisons

In a wide-ranging study, the relative cost-effectiveness for 26 different HIV prevention interventions was estimated [160]. The authors found that in low-prevalence populations, the most cost-effective interventions were structural interventions, and in high-prevalence populations, interventions aimed at behavioural change were fairly cost-effective. Showing educational videos in STI clinics and raising alcohol taxes were among the most cost-effective prevention measures, whereas school-based education was one of the least cost-effective measures [160]. Another study compared the cost-effectiveness of STI prevention interventions in the US targeting MSM, IDUs, and sexually active heterosexuals [161]. The main finding was that interventions for high-risk populations such as MSM were most cost-effective, as well as measures associated with the care continuum, whereas interventions centred on populations potentially at risk (versus those who could transmit HIV) were the least cost-effective [161].

In a systematic review on the four measures recommended by the US CDC (HIV testing, prevention with PLWH and their partners, condom distribution, and policy initiatives), researchers found that published evidence only indicates the cost-effectiveness of HIV testing [162]. A more recent review indicated that cost-effectiveness can be considered established for interventions such as TasP, PrEP, testing, condoms, circumcision, behavioural interventions, and the prevention of MTCT [163].

##### Comparing Interventions in Specific Populations

The seven studies in this category revealed that combinations of interventions targeted at specific populations can be cost-effective, but that interventions for high-risk populations, such as MSM, are the most cost-effective. In contrast, for low-prevalence populations, the most cost-effective interventions were structural interventions, as they are generally cheaper.

Comparing different policy scenarios in the US, one study argued that an expansion of testing and prevention services for *PLWH* would be not only cost-effective, but also necessary to achieve national goals, in addition to scaling up coverage of HIV care and treatment [164]. Given the growing number of older PLWH in high-income countries, age-appropriate strategies should be adopted, whereas testing and other prevention efforts, such as education and outreach, are needed for older adults [165].

Because *IDUs* have a high prevalence and incidence of HCV infections, a systematic review was performed focusing on the cost effectiveness of prevention, screening and treatment [166]. Many cost-effectiveness ratios were below USD 100,000/QALY gained. Evaluating different programmes for IDUs in the context of the opioid epidemic in the US, another study concluded that investments in combined prevention programmes could markedly lower the risk of HIV transmission and enhance health outcomes [167]. In contrast to PrEP, other approaches such as opioid agonist therapy, needle exchange, and test and treatment are considered cost-effective if implemented alone or in combination. Similarly, a UK study on IDUs found that a combination of outreach, testing and treatment of hepatitis C within drug treatment units presented a cost-effective option, with an estimated incremental cost of GBP 1029/QALY gained [168].

For *imprisoned MSM*, one publication reported different scenarios and found that a screening, treatment, and condom provision intervention could avert many STIs at low costs and would be cost-saving if it results in moderately higher condom use in sexual activity after being released from prison [169].

Last, a systematic review of economic evaluations of one-to-one interventions for *young adults* to reduce STIs, as well as unplanned pregnancies, concluded that most of the studies, typically concerning testing or counselling strategies, revealed either cost-effective or even cost-saving outcomes in some instances [170].

## Discussion

This scoping review shows that economic evaluations of interventions in the field of STI/HIV prevention are commonly based on mathematical modelling analyses, are frequently conducted in a US context, focus primarily on HIV, often target MSM as the main risk group, and a substantial portion of the studies evaluate testing strategies. This evidence suggests that most of the prevention interventions under investigation were cost-effective; only a few publications reported measures to not be cost-effective. Cost-effectiveness estimates, however, should be interpreted with caution given the heterogeneity of approaches. Moreover, cost-effectiveness thresholds are not always defined and differ significantly, for example, with a willingness-to-pay threshold of USD 150,000 per QALY gained reported in a US study [138], or EUR 20,000 per QALY in the Netherlands [133]. The results of economic evaluations further depend heavily on model assumptions, and input parameters vary widely across studies. Although we observed the increased use of a standardised economic evaluation methodology, especially in HIV prevention research, outcomes per type of intervention are not always assessed with standardised methods.

### Evidence Synthesis for Policy-Making

Although the included studies were very heterogeneous, some conclusions and general trends can be discerned.

First, structural approaches, such as needle-syringe exchange and condom distribution programmes, are economically efficient. In low-prevalence populations, structural interventions are the most cost-effective measure.

Second, behavioural interventions focusing on counselling and aiming to reduce risk behaviour can be cost-effective, especially if targeted toward high-risk groups such as IDUs or MSM. School-based interventions can be cost-effective, but the included studies found only a modest effect on sexual risk-taking behaviour or infection rates of such interventions.

Third, the substantial body of evidence on biomedical strategies supports the expansion of screening strategies, which would lead to a decrease in morbidity and mortality, as well as a reduction in the transmission of HIV/STIs. Even though the debate of whom to test and how frequently remains contested, study results support repeated testing for high-risk groups and one-time screenings for low prevalence populations. PrEP appears to be a cost-effective HIV prevention measure for high-risk individuals among the population of MSM, especially if a long time horizon is considered and if drug prices decline. Studies discussing treatment as prevention show it to be a crucial strategy in the field of HIV/STI prevention, leading not only to individual and public health gains, but also economic benefits.

### Limitations

This study has several limitations. First, due to its broad perspective, our scoping review includes studies that are heterogeneous with regard to targeted groups, settings, outcomes, and interventions assessed. A drawback is that we were unable to conduct a meta-synthesis and compare effect sizes across studies. For instance, some studies differentiated between cost-effective and cost-saving interventions, whereas others did not. Since we did not use a common threshold of cost-effectiveness, it is difficult to generalise the results, as the definitions of cost-effectiveness varied substantially between papers. Second, due to the large number of studies included, we were not able to assess the quality of the individual studies in detail. This is not mandatory for a scoping review methodology [7]; however, assessing the quality of the original sources would enhance the validity of the findings. Mathematical modelling analyses, which account for the majority of the studies, depend mostly on secondary data. Last, this review was restricted to studies published in English, which may have caused bias in excluding articles written in other languages.

### Knowledge Gaps and Implications for Further Research

This review allowed us to identify a number of weaknesses and research gaps.

An initial drawback is that the robustness of the cost-effectiveness findings strongly depends on the assumptions and thus on the available data. For instance, behavioural outcomes are more difficult to measure, and baseline data are often missing. Moreover, data on the exact allocations of costs were not available in many publications, which is why it is difficult to compare results, as costs may vary significantly if measures are applied in contexts other than the ones studied.

Second, we must consider possible bias in the included studies. For instance, only a few studies reported interactions between different prevention strategies or discussed how their results have been used in weighing policy alternatives. It is also often unclear how they isolated the impact of a particular intervention under investigation. In addition, not all studies were transparent on potential conflicts of interest, which could be an issue, especially for biomedical interventions. For instance, six studies on PrEP (out of 23) reported no funding information.

Third, many studies found a positive cost-effectiveness ratio since the costs of measures are contrasted with the lifetime treatment costs of HIV, which are generally still very high. With the availability of generic drugs, however, this assessment may change in the future, possibly leading to less favourable cost-effectiveness ratios. Whether a specific intervention becomes more or less cost-effective therefore also depends on advancements in the medical treatment of STIs and HIV/AIDS. Another aspect is the discounting of future health benefits, whereby most studies discounted health benefits at the same rate as costs, i.e. at 3%. Last, future economic evaluations should focus on the empirical assessment of programmes as focal aspects. Cross‐study comparability could be enhanced by clearly reporting and reflecting on contextual and biological factors. This would also be vital regarding the transferability of the results from one setting to another.

An important gap in the literature, highlighted in earlier studies, concerns combinations of interventions, which are crucial in the field of HIV/STI prevention [163]. Cost-effectiveness analyses of interventions at later stages of the sequence of HIV care, such as linkage and retention in HIV/AIDS care, were not examined much compared to testing strategies. Further, there is much more evidence on biomedical interventions than on structural approaches or on interventions aimed at behavioural change. In addition, interventions that focus on digital options are absent in the literature. In recent years, eHealth has become a critical topic, which opens up a promising research agenda for future economic evaluations. Last, services for specific populations have been understudied, such as transgender people, who have a very high burden of HIV [171], sex workers, or foreign-born migrants at higher risk. Although earlier reviews have pointed out that the field is very unbalanced and programmes for significant populations in the HIV epidemic have been understudied [1], there is still an unequal distribution regarding research evidence.

## Supplementary Information

Below is the link to the electronic supplementary material.

Supplementary file1 (DOCX 76 kb)

Supplementary file2 (DOCX 36 kb)

Supplementary file3 (DOCX 425 kb)

## Data Availability

The database with literature coding can be obtained from the corresponding author.
