# Supplementary material for: The Cost-Effectiveness of HIV/STI Prevention in High-Income Countries with Concentrated Epidemic Settings: A Scoping Review
Source: AIDS Behav. 2022 Jan 15;26(7):2279–98. doi: 10.1007/s10461-022-03583-y (PMC9163023; doi:10.1007/s10461-022-03583-y)
Supplement: Supplementary file 1 — Supplementary file1 (DOCX 76 kb) [file 10461_2022_3583_MOESM1_ESM.docx]

|  |
| --- |
|  |
|  |

**Research Protocol**

**Written in line with PRISMA-P 2015 Statement^[[1]](#footnote-1)^**

**Date of the protocol: January 2019 [update January 2021]**

# ADMINISTRATIVE INFORMATION

1. **Title:** The cost-effectiveness of HIV/STI prevention in high income countries with concentrated epidemic settings. A scoping review.
2. **Registration:** To be registered in PROSPERO [no registration due to change in registration rules after onset].
3. **Authors:** Palmo Brunner [PB], Karma Brunner [KB], Daniel Kübler [DK]

Contact: Daniel Kübler (Daniel.Kuebler@ipz.uzh.ch)

Contributions: DK is the project leader. DK and PB conceived the study and designed the search strategy. All authors will participate in its implementation and contribute to identifying eligible studies. PB and KB will be the first and second reviewer and will conduct data extraction. DK is the third reviewer, who will be consulted in case of conflict or uncertainty. PB and DK will contribute to data interpretation and write the article. All authors will read and approve the final manuscript.

1. **Amendments:** Important protocol amendments will be recorded and included in dissemination. If we need to amend the protocol, we will describe the change and give an explanation of the rationale.
2. **Support:** This study was supported and funded by the Medical Services of the canton of Zurich and the city of Zurich, Switzerland. Role of the sponsor/funder: Representatives of the sponsors were part of the project’s advisory group alongside other experts and researchers. The funders had control over neither design nor implementation of this study, nor over the interpretation or publication of the results, which were the sole responsibility of the authors.

# INTRODUCTION

1. **Rationale**

It is well recognized that sexually transmitted infections (STIs) are a significant public health issue – first and foremost HIV/AIDS. The UNAIDS global statistics^[[2]](#footnote-2)^ reported that in 2018, more than 37.9 million people are globally living with HIV, whereas 1.7 million people became newly infected and 770 thousand people died due to AIDS-related diseases. Efforts to end the HIV epidemic and to combat sexually transmitted diseases, however, require extraordinary amounts of investments. Since total health spending increased worldwide (Dieleman et al. 2018), evidence of the cost-effectiveness of different interventions and programs is crucial. Selecting optimal allocation strategies are vital to achieving the highest benefits with the available resources. Policy makers thus need to balance the costs and benefits of numerous interventions when planning HIV prevention programs. Economic evaluations are therefore an important tool in support of the decision-making process by systematically quantifying and comparing the costs and outcomes of different interventions (Rudmik and Drummond 2013).

However, the growth of economic evaluation studies in the field of HIV prevention (Tran et al. 2019) make it hard for decision-makers to keep on track and maintain an overview. A systematic review summarizes the results of available studies and provides a synthesis as well as an appraisal of the knowledge in a particular field (Møller and Myles 2016). Recommendations for policymaking thus may be informed on basis of such a systematic review.

1. **Objectives**

The main aim of this study is to systematically review the current state of economic evaluations in the field of HIV/STI prevention. The objective of the study is twofold: First, to summarize the evidence regarding the cost-effectiveness of different prevention programs and interventions in order to support policymaking; and second, to determine the research landscape and identify potential shortcomings as well as research gaps. To this end, the proposed systematic review will answer the following questions:

- *What is the evidence regarding the cost-effectiveness of preventive interventions in the field of HIV/STI?*
- *What are the trends and patterns in economic evaluation studies in the field of HIV/STI prevention?*

# METHODS

1. **Eligibility criteria**

Inclusion criteria according to PICO guidelines, however, we deliberately want to review a wide range of studies in the field of HIV/STI prevention in order to have a comprehensive synthesis of existing evidence.

PICO: We include economic evaluations of preventive interventions or programs (I) targeting the general population, risk groups, or already infected people (P). Comparison between different interventions or on/off comparison (C). Different outcomes of HIV/STI prevention (O) such as averted infections, behaviour change, and quality adjusted life years.

Context: Studies focusing on western countries with concentrated epidemic settings will be included in order to have a comparable context regarding not only financial resources available, but also differences in transmission types.

Time: Studies after 1998, when the first combination drug therapy became available, are eligible.

Language: We will include articles reported in English, German and French. [Due to low number of studies published exclusively in German or French, only English language publications were included in the end.]

Design: no restrictions. Any design of economic evaluations.

Exclusion: The focus clearly lies on HIV/STI prevention and not on treatment in general. Only studies explicitly researching interventions in the context of disease prevention will be included. Studies focusing simply on the effectiveness of specific interventions but not taking into account monetary aspects will be excluded.

1. **Information sources**

The search will employ topic-based strategies designed for each database from inception to 30^th^ September 2019. The following databases will be searched electronically.

- Web of Science;

- Scopus;

- NHS EED (National Institute for Health Research Economic Evaluation Database);

- ERIC (Education Resources Information Center);

- Cochrane library;

- Campbell library;

- Cost effectiveness analysis registry;

[It turned out that Web of Science and Cost-effectiveness analysis registry were the most comprehensive databases for our purposes. Only these two databases were considered in the end.]

Additional relevant studies proposed by the advisory group as well as grey literature can be included. [In the end, there were not additional studies proposed by the advisory board.]

1. **Search strategy**

The search strategy will be tailored to each database and reported in the review. It is based on the following three main concepts: HIV and/or STI, prevention, and economic evaluation.

Example of string

HIV OR HIV/AIDS OR AIDS OR HIV/STI OR STI OR HIV/STD OR STD) AND prevent* AND (cost* OR spend*) AND (effective* OR consequence* OR utilit* OR benefit* OR efficiency OR (economic AND evaluation))

1. **Study records:**

**Data management:** Records will be managed through Citavi, a specific software for managing bibliographies. For data extraction, MAXQDA as well as Excel will be used.

**Selection process:** First, one reviewer, PB, using the a priori inclusion/exclusion criteria will screen titles and abstracts (where available) for eligibility. Full papers will be obtained for those that appear relevant. Second, a second reviewer, KB, will read the full text of each article and check for inclusion again. Exclusion at this stage may resolved by discussion between the two reviewers (PB, KB), a third person (DK) being available for conflicts. We will report the reasons for excluding studies at this stage. A flow diagram will provide an illustration of the whole process.

**Data collection process:** Data will be abstracted by one reviewer (KB) using a standardized form. Therefore, a codebook was developed which was piloted and refined by all reviewers prior to abstraction. A second reviewer (PB) will independently check the data for consistency and clarity. If there is uncertainty or conflict on any data element, a third reader is consulted (DK).

1. **Data items**

For all included publications, the following data will be abstracted: formal criteria, focus of the study, target group, intervention, outcome, study design, and relevant details regarding cost effectiveness as well as quality appraisal.

1. **Outcomes and prioritization**

Primary and secondary outcomes will be reported. Discussion with the project’s advisory group will enable the prioritisation of a policy-relevant subset of studies if necessary.

1. **Risk of bias in individual studies**

Risk of bias on a study-level will be assessed with consideration of selective reporting, incomplete data, possible cofounders, selection bias, dropouts and blinding for RCTs. This assessment will be incorporated in the synthesis.

1. **Data synthesis**

The results will be summarized in a narrative synthesis with a quantitative overview; seminal studies may be analyzed and discussed qualitatively as well. It is unlikely that a meta-analysis will be possible based on heterogeneity between the included studies (diversity in populations, interventions, outcomes, or design).

1. **Meta-bias(es)**

The synthesis will also provide a critical discussion of the relationships within and between studies to provide an overall assessment of the robustness of the evidence.

1. **Confidence in cumulative evidence**

Limitations at review-level (e.g., incomplete retrieval of identified research, reporting bias) will be discussed.

**References**

Dieleman, Joseph L.; Haakenstad, Annie; Micah, Angela; Moses, Mark; Abbafati, Cristiana; Acharya, Pawan et al. (2018): Spending on health and HIV/AIDS: domestic health spending and development assistance in 188 countries, 1995–2015. In *The Lancet* 391 (10132), pp. 1799–1829. DOI: 10.1016/S0140-6736(18)30698-6.

Møller, A. M.; Myles, P. S. (2016): What makes a good systematic review and meta-analysis? In *British journal of anaesthesia* 117 (4), pp. 428–430. DOI: 10.1093/bja/aew264.

Rudmik, Luke; Drummond, Michael (2013): Health economic evaluation: important principles and methodology. In *The Laryngoscope* 123 (6), pp. 1341–1347. DOI: 10.1002/lary.23943.

Shamseer, Larissa; Moher, David; Clarke, Mike; Ghersi, Davina; Liberati, Alessandro; Petticrew, Mark et al. (2015): Preferred reporting items for systematic review and meta-analysis protocols (PRISMA-P) 2015: elaboration and explanation. In *BMJ (Clinical research ed.)* 350, g7647. DOI: 10.1136/bmj.g7647.

Tran, Bach Xuan; Nguyen, Long Hoang; Turner, Hugo C.; Nghiem, Son; Vu, Giang Thu; Nguyen, Cuong Tat et al. (2019): Economic evaluation studies in the field of HIV/AIDS: bibliometric analysis on research development and scopes (GAPRESEARCH). In *BMC health services research* 19 (1), p. 834. DOI: 10.1186/s12913-019-4613-0.

1. “The Preferred Reporting Items for Systematic Reviews and Meta-Analyses Protocols”, see Shamseer et al. 2015. [↑](#footnote-ref-1)
2. <https://www.unaids.org/en/resources/fact-sheet> [↑](#footnote-ref-2)
