# Supplementary material for: The Cost-Effectiveness of HIV/STI Prevention in High-Income Countries with Concentrated Epidemic Settings: A Scoping Review
Source: AIDS Behav. 2022 Jan 15;26(7):2279–98. doi: 10.1007/s10461-022-03583-y (PMC9163023; doi:10.1007/s10461-022-03583-y)
Supplement: Supplementary file 2 — Supplementary file2 (DOCX 36 kb) [file 10461_2022_3583_MOESM2_ESM.docx]

**S2: Codebook**

| **Formal criteria** | | | **Variable** |
| --- | --- | --- | --- |
| Author | Last Name of 1st author | | Author |
| Year of Publication (YoP) | Format: YYYY (auch: YYYYa) | | YoP |
| Country of affiliated institution (1st author) | Shortcut English (eg: Germany = GER, France = FRA, USA, CH, Australia = AUS, New Zealand = NZ etc.) | | Country_author |
|  | | | |
| **Focus of the Study** | | | **Variable** |
| **Country / Region of evaluation** | 1 Western Europe  2 North America  3 Australia, NZ  4 International  90 other (🡪 Specify in “Country”)  0 no information/unknown/not applicable  99 unclear to coder | | CountryRegion |
|  | Country Name | string | Country |
| **General focus** | 1 HIV/AIDS  2 STIs in general (or two or more)  3 HIV and other STI  4 Specific STI (=> GenFocSTI)  90 other  0 no information/unknown/not applicable  99 unclear to coder | | GenFoc |
| **Focussed STI** | ***If GenFoc=1***  1 HIV  2 AIDS  ***If GenFoc=4***  3 Syphilis(Lues)  4 Gonorrhea  5 Chlamydia  6 Hepatitis B (HBV) and C (HCV)  90 other  0 no information/unknown/not applicable  99 unclear to coder | | GenFocSTI |
| **Target groups** | 1 General public  2 Young adults, youth  3 MSM  4 Transsexual, transgender  5 Migrants  6 Ethnically defined groups  7 FSW  8 MSW  9 Clients of FSW / MSW  10 IDU (injecting drug user)  11 Prisoners, Inmates  12 PLWH (people living with HIV)  13 Partners of HIV positive people  14 Pregnant women  66 more than one target group  90 other  0 no information/unknown/not applicable  99 unclear to coder | | TarGroups |
| **Group specification** | *If TarGroup=66* Numbers  *If TarGroup=90* String | | GroupSpec |

| **Method / Study Design** | | | | |
| --- | --- | --- | --- | --- |
| **Study design**  (to measure the effect/causality) | Before/after measurements | 0 no / 1 yes | | StuDes_befaft |
|  | Control and experimental group | 0 no / 1 yes | | StuDes_contrexp |
|  | Randomized procedure | 0 no / 1 yes | | StuDes_random |
|  | Mathematic Modelling | 0 no / 1 yes | | StuDes_mathmod |
|  | Review | 0 no / 1 yes | | StuDes_review |
| **Period of data collection** | Start Year of data collection | YYYY | | Period_data_coll_start |
|  | End Year of data collection | YYYY | | Period_data_coll_end |
| **Time perspective** | 1 retrospective  2 prospective  3 cross-sectional  90 other  0 no information/unknown/not applicable  99 unclear to coder | | | Time_persp |
| **Data collection** | 1 primary data  2 secondary data  3 both  90 other  0 no information/unknown/not applicable  99 unclear to coder | | | Data_collect |
| **Discounting rate** | X% | | 0 no  1 dynamic  2 non dynamic  90 yes, other  99 unclear | Eff_discounting |
| **Estimations** | 0 no / 1 yes | |  | Eff_estimations |
| **Modelling** | Have effects been modelled (eg based on other data) | |  | Eff_modelling |

| **Intervention** | | |
| --- | --- | --- |
| **Type of interventions** | 1 Sex education (school based, youth oriented projects)  2 Mass media campaign  3 Counseling / information (individual or group)  4 Vaccination  5 Outreach and mobilization (on the street, in the community)  6 Screening and/ or testing  7 VCT (Voluntary counseling and testing)  8 Condom availability / distribution (also female)  9 Needle exchange, Syringe program, harm reduction  10 HIV treatment, ART, HAART  11 HIV PrEP  12 HIV PEP  13 STI treatment  14 Partner notification  15 MTCT prevention (mother-to-child transmission of HIV)  66 more than one intervention  90 other  0 no information/unknown/not applicable  99 unclear to coder | intv |
| **PREP** | *If intv =11* 0 PrEP in general / 1 PREP on demand / 2 PREP (daily) | prep |
| **Intervention specification** | *If intv=66* Numbers  *If intv=90* String | IntvSpec |

| **Outcome / Effect** | | | |
| --- | --- | --- | --- |
| **Primary outcome**  (Primary focus of the study) | 1 infections prevented (HIV, STI)  2 decrease in risk behaviour  3 increase in prevention knowledge  4 DALYs gained (disability adjusted life years)  5 QALYs gained (quality adjusted life years)  6 Life years gained (LYG)  7 Secondary infections averted  8 Averted costs of (future) treatment  9 deaths averted  10 STI case treated  11 change in prevalence  12 change in testing behavior  13 early infections detected  66 more than one outcome (-> no primary focus, equal weight)  90 other (build new category)  0 no information/unknown/not applicable  99 unclear to coder | | Prim_Outcome |
| **Additional outcome** | 1 infections prevented (HIV, STI)  2 decrease in risk behaviour  3 increase in prevention knowledge  4 DALYs gained (disability adjusted life years)  5 QALYs gained (quality adjusted life years)  6 Life years gained (LYG)  7 Secondary infections averted  8 Averted costs of (future) treatment  9 deaths averted  10 STI case treated  11 change in prevalence  12 change in testing behavior  13 early infections detected  90 other (build new category)  0 no information/unknown/not applicable  99 unclear to coder | | Sec_Outcome |
| **Primary Effect** | 1 positive effective  2 No effect  0 no information/unknown/not applicable  99 unclear to coder | | Prim_effect |
| **Additional Effect** | 1 positive effective  2 No effect  0 no information/unknown/not applicable  99 unclear to coder | | Add_effect |
| **Monetarising** | Treatment costs  QALY  DALY  WTP (willingness-to-pay)  Mortality/morbidity  Productivity gains | 0 no  1 yes | Treatm_costs |
|  |  |  | QALY |
|  |  |  | DALY |
|  |  |  | WTP |
|  |  |  | MortMorb |
|  |  |  | ProdGain |

| **Cost effectiveness** | | | | |
| --- | --- | --- | --- | --- |
| **Perspective**  (regarding type of cost collected) | 1 (health) provider *(omits costs incurred by private consumers)*  2 societal *(costs incurred by all members of society -> private & public)*  3 public *(only costs of public sector)*  4 private sector or private consumers  90 other (-> string)  66 more than one  0 no information/unknown/not applicable  99 unclear to coder | | | Perspective_costs |
| **Time horizon** | Number xy lifetime string  66 more than one time scenario  0 no information/unknown/not applicable  99 unclear to coder | | | Time_horizon |
| **Costs** *= Monetary valuations of input to produce outcome* | Are direct costs listed/explicitly named? | | 0 no  1 yes  2 only discussed | Direct_costs |
|  | Are indirect costs listed/explicitly named? | |  | Indirect_costs |
|  | Are *intangible* costs listed/explicitly named? | |  | Intangible_costs |
| **Intv costs overall** | Are the total costs of the intervention(s) named? | | 0 no  1 yes  2 yes with details | Overall_costs |
| **Cost per outcome** | Costs estimated  ***If cost=yes***  Cost per primary outcome  Cost per secondary outcome | 0 no / 1 yes  99 unclear  xy DollarPPP  xy DollarPPP | | cost  Cost_pri_outcome  Cost_sec_outcome |
| **Basis of comparison /**  **of economic assessment** | 1 on/off (also if >1 intv but as programme/mix/a whole)  2 one type in diff. variations  3 more than one type of comparisons  4 (prevention interventions ⬄) treatment  90 other  0 no information/unknown/not applicable  99 unclear to coder | | | Basis_comparison |
| **Type of economic evaluation**  (as named by study) | 1 Cost-effectiveness analysis (natural unit)  *CEA takes into account one single effect measured in natural units (e.g. infections prevented, decrease in risk behaviour, increase in prevention knowledge).*  2 Cost-consequences analysis (natural units)  *CCA takes into account several (= more than one) effects measured in natural units (e.g. infections prevented and life years gained).*  3 Cost-utility analysis (health utility, e.g. QALY)  *CUA adds the value or worth of a specific effect for an individual or for society. E.g. it asks not only what it costs for alternative intervention to extend a person’s life by two years, but also confronts the question of how much utility an increase in life has when it is accompanied by pain or disability (often used: QALY)*  4 Cost-benefit analysis (money)  *CBA translates costs and consequences into the same metric (usually monetary), so that they can be expressed in a simple term, such as ratio of benefits to costs.*  90 other (build new category)  0 no information/unknown/not applicable  99 unclear to coder | | | Type_econeval |
| **Cost effectiveness** | 1 not cost effective  2 cost effective  3 cost saving  4 cost effective / cost saving only under certain conditions  66 more than one outcome (-> no primary focus, equal weight)  0 no information/unknown/not applicable  99 unclear to coder | | | Cost_eff |

| **Quality Assessment** | | | |
| --- | --- | --- | --- |
| **Sensitivity Analysis** | Performed? | 0 no / 1 yes | sensitivity |
| **Limitations mentioned regarding** | Costs  Effects / Outcome  General / study level | 0 no / 1 yes | Cost_limit  Effects_limit  General_limit |
| **Risk of Bias** | 0 low  *(Blinding for RCT, selective reporting, incomplete data*  1 high *confounders, selection bias, drop-outs etc.)*  99 unclear | | bias |
| **Funding** | 0 no funding details reported  1 funding / sponsorship reported  2 (non-)involvement of funding / sponsorship discussed  99 unclear | | funding |
