# Supplementary material for: The Cost-Effectiveness of HIV/STI Prevention in High-Income Countries with Concentrated Epidemic Settings: A Scoping Review
Source: AIDS Behav. 2022 Jan 15;26(7):2279–98. doi: 10.1007/s10461-022-03583-y (PMC9163023; doi:10.1007/s10461-022-03583-y)
Supplement: Supplementary file 3 — Supplementary file3 (DOCX 425 kb) [file 10461_2022_3583_MOESM3_ESM.docx]

| STRUCTURAL APPROACHES (15 ARTICLES) | | | | |
| --- | --- | --- | --- | --- |
| **Reference** | **Target group(s) / population** | **Country** | **Design / Model reported** | **Intervention(s)** |

| **Broad policy initiatives and national programs (3)** |
| --- |

| **(Chesson 2006)** | General population | USA | Random-mixing epidemiological model & steady-state analysis | National gonorrhoea control program |
| --- | --- | --- | --- | --- |
| **(Dauner et al. 2008)** | Community based organizations (CBO) | USA | Threshold analyses | National capacity-building program for HIV prevention organizations |
| **(Choi et al. 2016)** | General population | Canada | Macro-level evaluation | Community-Based HIV Prevention Programs in Ontario (Education, outreach, campaigns, adherence, social support, needle syringe programs) |

| **Needle and Syringe Program (NSP) (4)** |
| --- |

| **(Pollack 2001)** | IDUs | USA | Random-mixing epidemiological model & steady-state analysis | NSP to reduce HCV incidence and prevalence |
| --- | --- | --- | --- | --- |
| **(Wodak and Cooney 2005)** | IDUs | Developed countries | Review | NSPs |
| **(Kwon et al. 2012)** | IDUs | Australia | Health economic analysis incorporating a mathematical model | NSPs |
| **(Sweeney et al. 2019)** | IDUs | UK | Cost-effectiveness analysis utilizing a dynamic transmission model | NSPs |

| **Condom Distribution Programs (CDPs) (7)** |
| --- |

| **(Bedimo et al. 2002)** | African Americans | USA | Cost-utility analysis | Louisiana condom social marketing program |
| --- | --- | --- | --- | --- |
| **(Holtgrave et al. 2012b)** | Women | USA | Retrospective economic evaluation (Cost-utility analysis) | Female condom distribution and education program |
| **(Sadler et al. 2017)** | Different target groups | UK | Economic model using a Bernoulli Process | CDPs for young people, black ethnic minorities, MSM and the general population |
| **(Gopalappa et al. 2012)** | PLWH | USA | Mathematic modelling | Early Linkage |
| **(Spaulding et al. 2013)** | HIV-infected releasees | USA | Mathematic modelling, cost-effectiveness analysis | EnhanceLink, a community transitional program for HIV-infected jail detainees |
| **(Jain et al. 2016)** | PLWH | USA | Mathematic modelling, cost threshold analyses | HIV linkage and retention in care programs |
| **(Maulsby et al. 2018)** | PLWH | USA | Mathematic modelling cost–utility analysis | HIV linkage, re-engagement and retention (LRC) in care programs |

| **Comparison of different interventions (1)** |
| --- |

| **(Cohen et al. 2006)** | Women | USA | Mathematic modelling (“Maximizing the benefits”) | Comparison of structural interventions: Condom availability, needle exchange & needle deregulation, alcohol taxes, mass media campaigns, street outreach, opinion leaders programs |
| --- | --- | --- | --- | --- |

| BEHAVIOURAL INTERVENTIONS (18 ARTICLES) |
| --- |

| **Reference** | **Target group(s) / population** | **Country** | **Design / Model reported** | **Intervention(s)** |
| --- | --- | --- | --- | --- |

| **Counselling (15)** |
| --- |

| **(Marseille et al. 2011)** | PLWH | USA | Cost-effectiveness model | Three types of counselling-based interventions in clinical settings at 13 sites: (1) clinical provider, (2) specialist, and (3) mixed services, |
| --- | --- | --- | --- | --- |
| **(Safren et al. 2015)** | PLWH (MSM) | USA | Commentary | Individually based behavioural interventions for MSM with uncontrolled virus |
| **(Kahn et al. 2001)** | MSM (young) | USA | Retrospective cost-effectiveness analysis | Mpowerment Project community-level intervention |
| **(Herbst et al. 2007)** | MSM (adult) | Inter-national | Systematic Review | Individual-, group-, and community-level behavioural interventions intended to reduce the risk of acquiring HIV |
| **(Zaric et al. 2008)** | MSM (on HAART) | USA | Dynamic compartmental model | Counselling intervention given prior to initiation of HAART and before all changes in drug regimens, combined with phone-in support while on HAART. |
| **(Pinkerton et al. 2000)** | IDUs (out-of- treatment) and their sex partners | USA | Mathematical modelling | National AIDS Demonstration Research (NADR) program to reduce s risks behaviour: 8 different interventions, different research sites |
| **(Tuli et al. 2005)** | IDUs (HIV-seropositive heterosexual) | USA | Mathematical modelling | INSPIRE designed to reduce risky sexual / needle-sharing behaviours: 10 education sessions (seven small- group, one peer group volunteer activity, two individual) |
| **(Ruger et al. 2014)** | IDUs (women) | USA | Standard methods of cost-effectiveness analysis | (1) Standard intervention (SI); (2) SI plus a well woman exam (WWE); and (3) SI, WWE, plus 4 educational sessions (4ES). |
| **(Song et al. 2015)** | IDUs (HIV infected) | USA | Dynamic HIV transmission model | Comparison of the Holistic Health Recovery Program for HIV+ (HHRP+) with abbreviated Holistic Health for HIV (3H+) Program |
| **(Burgos et al. 2010)** | FWSs | Mexico–US Border Region | Markov model | Brief behavioural intervention to reduce incidence of HIV and STIs among FSWs: (1) once-only (2) annually |
| **(Johnson et al. 2013)** | Young men being released from prison | USA | Threshold analysis | Project START: (1) single-session intervention and a (2) multi-session risk-reduction intervention |
| **(Johnson-Masotti et al. 2000)** | Adults with severe mental illness | USA | Cost-utility analysis | Cognitive-behavioural HIV risk reduction interventions: (1) a single one-on-one intervention; (2) a multi-session small-group intervention; and (3) a multi-session small-group intervention and advocacy training |
| **(Pinkerton et al. 2001)** | Mentally ill adults | USA | Cost-utility analysis | Small-group interventions that focus on sexual communication, condom use skills, and motivation to practice safer sex |
| **(Johnson-Masotti et al. 2005)** | Women | USA | Mathematical modelling | A multi-site community-level HIV prevention trial in 5 U.S. cities for women living in low-income housing developments |
| **(Chesson 2007)** | General population | USA | Cost-effectiveness analysis (and deterministic, compartmental model of STI transmission) | Behaviour-change prevention programme for STI initiated at various stages of an STI epidemic (hypothetical public awareness campaign delivered through the media to increase the use of condoms) |

| **School-based interventions (3)** |
| --- |

| **(Wang et al. 2000)** | High school students | USA | Mathematic Modelling with Bernoulli model | School-based sexual risk intervention |
| --- | --- | --- | --- | --- |
| **(Shepherd et al. 2010)** | Young people | Inter-national and UK | Review (and Bernoulli statistical model to describe the probability of STI infection) | Schools-based skills building behavioural interventions |
| **(Cooper et al. 2012)** | Teenagers aged 15 | UK | Cost-Effectiveness analysis | School-based (1) teacher-led and (2) peer-led behavioural interventions |

| BIOMEDICAL INTERVENTIONS (101 ARTICLES) |
| --- |

| **Reference** | **Target group(s) / population** | **Country** | **Design / Model reported** | **Intervention(s)** |
| --- | --- | --- | --- | --- |

| **Testing and Screening (56)** |
| --- |

| General Population (16) | | | | |
| --- | --- | --- | --- | --- |
| **(Bos et al. 2001)** | General population | Netherlands | Bernoulli model | Universal HIV screening of patients attending a STI clinic |
| **(Paltiel et al. 2005)** | General population / Different target groups | USA | Computer simulation model | HIV screening and treatment to compare routine, voluntary recommend routine HIV counselling, testing, and referral with current practice |
| **(Walensky et al. 2005)** | General population | USA | cost-effectiveness analysis using a computer simulation model | Counselling, testing, and referral for patients in hospitals |
| **(Paltiel et al. 2006)** | General population | USA | Cost-effectiveness analysis | One-time and increasingly frequent voluntary HIV screening of all adults |
| **(Holtgrave 2007a)** | General population | USA | Basic methods of scenario & cost-effectiveness analysis | Opt-out HIV testing (testing without the need for risk assessment and counselling) in all health care encounters |
| **(Walensky et al. 2007)** | General population | USA | Review | Testing for all persons aged 13–64 years in health care settings |
| **(Gift et al. 2008)** | Men | USA | compartment-based transmission model | Screening men for chlamydia compared with alternative interventions |
| **(Deogan et al. 2010)** | General Population | Sweden | Cost-effectiveness model | “Chlamydia Monday”: information and increased availability to testing, treatment and contact tracing. The intervention begins with a publicity campaign |
| **(Martin et al. 2010)** | General population | USA | Simulation model | Expanded HIV screening |
| **(Dowdy et al. 2011)** | General population | USA | Cost-utility analysis | Rapid HIV screening program in an urban ED |
| **(Haukoos et al. 2013)** | General population | USA | Prospective cohort study nested in a larger quasi-experiment | Compare programmatic costs of (1) no targeted opt-out rapid HIV screening (2) physician-directed diagnostic rapid HIV testing in an urban EDs |
| **(Yazdanpanah et al. 2013)** | General population | Portugal | Model-based assessment | Routine HIV Screening (1) Current HIV target screening (2) On-demand screening |
| **(Hsu et al. 2013)** | General population | USA | Review | Routine HIV screening programs in EDs |
| **(Thanh et al. 2017)** | Women | Canada | Dynamic Markov model | (1) universal urogenital-only screening (2) additional selected (exposure-based) rectal screening (3) additional universal rectal screening in STI clinics |
| **(Baggaley et al. 2017)** | General population | UK | Cost-effectiveness analysis | Screening for HIV in general practices (primary care) |
| **(Deuffic-Burban et al. 2018)** | General population / Different target groups | France | Markov model | Expanded HCV screening |
| Youth (9) | | | | |
| **(Wang et al. 2002)** | Youth | USA | A decision-analysis model | A high school-based STI screening program to detect chlamydia and gonorrhoea |
| **(Hu et al. 2004)** | Young women | USA | State transition simulation model; cost-effectiveness analysis. | Different Chlamydia screening strategies |
| **(Aledort et al. 2005)** | Young women | USA | State-transition Markov model | Gonorrhea screening in urban emergency departments |
| **(Adams et al. 2007)** | Youth | UK | A stochastic, individual based, dynamic sexual network model combined with a cost effectiveness model | Chlamydia testing |
| **(Vries et al. 2008)** | Youth | Netherlands | Dynamic model | Chlamydia trachomatis screening with different frequencies |
| **(Wit et al. 2015)** | Youth | Netherlands | Mathematical modelling | Six Chlamydia screening scenarios |
| **(Gillespie et al. 2012)** | Youth | UK | Prospective cost analysis | Chlamydia screening in different healthcare settings |
| **(Looker et al. 2015)** | Youth | UK | Compartmental deterministic dynamic model | Chlamydia testing |
| **(Suijkerbuijk et al. 2018a)** | Youth | Netherlands | Economic evaluation | STI Testing |

| Partners (3) | | | | |
| --- | --- | --- | --- | --- |
| **Reference** | **Target group(s) / population** | **Country** | **Design / Model reported** | **Intervention(s)** |
| **(Shrestha et al. 2009)** | High index partners | USA | Cost-effectiveness analysis Using microcosting methods | Partner counselling and referral services to HIV-infected index patients and their partners |
| **(Rahman et al. 2015)** | Clients in STD clinics and partners | USA | Cost-effectiveness analysis based on an experimental basis | Telephone based partner notification |
| **(Nichols et al. 2015)** | MSM and partners | Netherlands | Mathematical modelling | Partner notification program |
| MSM (8) | | | | |
| **(Vriend et al. 2013)** | MSM (in care) | Netherlands | Transmission model combined with economic analysis | Screening for anorectal chlamydia at HIV treatment centres (1) Once-yearly or (2) twice-yearly |
| **(Chesson et al. 2013)** | MSM | USA | Markov state-transition model | Screening MSM who have a current or recent history of rectal Chlamydia trachomatis (CT) and Neisseria gonorrhoea (GC) |
| **(Chesson et al. 2016)** | MSM | USA | Exploratory Modelling Analysis | Syphilis Screening |
| **(Zwart et al. 2019)** | MSM | Netherlands | Modelling Study | Different testing strategies for anogenital gonorrhoea |
| **(Harte et al. 2011)** | MSM | UK | Economic Evaluation | Recalling MSM diagnosed as having a bacterial sexually transmitted infection for HIV re-screening |
| **(Juusola et al. 2011)** | MSM | USA | Dynamic model of HIV transmission and progression | Different HIV testing strategies |
| **(Hoenigl et al. 2016)** | MSM | USA | Cost-effectiveness and transmission analysis | Three community-based acute HIV infection (AHI) testing algorithms compared to HIV antibody testing alone |
| **(Zulliger et al. 2017)** | MSM | USA | Cost-Utility Analysis | MSM Testing Initiative (MTI) to newly diagnose HIV among MSM and link them to medical care |
| Other risk groups (10) | | | | |
| **(Shrestha et al. 2010)** | Minority populations | USA | Economic Evaluation | Four community-based organizations implemented a social network strategy for HIV counselling and testing |
| **(Suijkerbuijk et al. 2018b)** | Foreign born migrants | Netherlands | Markov model | HBV & HCV screening program |
| **(Pottie et al. 2018)** | Migrant populations | International | Review | HIV testing strategies in migrant populations |
| **(Martin et al. 2019)** | Migrant populations | UK | Decision model based on a Markov approach | Hepatitis B virus case-finding among migrant populations born in countries with intermediate or high prevalence levels (≥2%) in a primary care setting |
| **(Varghese and Peterman 2001)** | Prisoners | USA | Decision model | HIV testing and counselling |
| **(Schackman et al. 2013)** | Substance abusers | USA | Cost-effectiveness analysis of a randomized trial | HIV Testing in Substance Abuse Treatment: (1) off-site testing referral (2) on-site rapid testing with information only (3) on-site rapid testing with risk reduction counselling |
| **(Schackman et al. 2015)** | Substance abusers | USA | decision analytic mode | rapid HCV testing and simultaneous rapid HCV and HIV testing in substance abuse treatment programs |
| **(Shrestha et al. 2011)** | Transgender communities | USA | Cost-Effectiveness analysis | Rapid HIV testing |
| **(Wilson et al. 2010)** | Sex workers | Australia | A simple mathematical transmission model | Testing policy versus the health benefits of averting the transmission of HIV, syphilis, chlamydia and gonorrhoea to clients. |
| **(Holtgrave 2007b)** | High-risk communities | USA | Standard methods of scenario and cost-effectiveness analysis | HIV counselling and rapid testing in high-risk communities |

| Comparison of testing strategies and target groups (10) | | | | |
| --- | --- | --- | --- | --- |
| **Reference** | **Target group(s) / population** | **Country** | **Design / Model reported** | **Intervention(s)** |
| **(Heumann et al. 2001)** | Three high-risk groups: (A) young MSMs (B) older MSMs (C) IDUs | USA | Cost-effectiveness analysis | HIV prevention referrals to high-risk seronegatives receiving antibody testing |
| **(Shrestha et al. 2008)** | Not specified | USA | Retrospective economic evaluation of the program | Rapid HIV Testing in Community-Based Organizations (CBOs) |
| **(Prabhu et al. 2011)** | Different target groups | USA | Progression and Transmission model | HIV screening in three settings, (1) STD clinics serving SMS (2) EDs (3) Settings where patients are likely to be diagnosed early  (4) Inpatient diagnosis based on clinical manifestations |
| **(Long 2011)** | (A) Aged 15-64 (B) IDUs & MSM (C) MSM only | USA | A dynamic HIV transmission model | HIV Screening via (1) Fourth-Generation Immunoassay or (2) Nucleic Acid Amplification Test |
| **(Helsper et al. 2012)** | (A) General Population (B) additional primary care support (C) hard drug users | Netherlands | Mathematic modelling | Screening for hepatitis C (3 different campaigns) |
| **(Lucas and Armbruster 2013)** | Low and High-risk individuals | USA | Deterministic mathematical model | (1) One-Time test for Low-risk individuals (2) Annual testing for high risk individuals |
| **(Long et al. 2014)** | (A) MSM (B) IDU (C) Individuals from HIV-endemic countries (D) Adults | UK | Dynamic compartmental model | Expanded HIV Testing |
| **(Castel et al. 2015)** | Patients in all health care settings vs. persons at high risk (e.g. IDUs, persons with multiple sexual partners, MSMs) | USA | Cost-effectiveness analysis | Routine vs. targeted testing at different sites (CBO, clinic, hospital) |
| **(Hutchinson et al. 2016)** | MSM and IDUs | USA | Mathematical model of HIV transmission | Frequent HIV rapid testing at different intervals |
| **(Li et al. 2018)** | Different target groups | USA | Cost-utility analysis | (1) Rapid HIV tests at clinical settings (2) Rapid HIV tests at CBOs (3) Partner Notification Services (PNS) program |

| **Treatment as Prevention TasP (9)** |
| --- |

| **Reference** | **Target group(s) / population** | **Country** | **Design / Model reported** | **Intervention(s)** |
| --- | --- | --- | --- | --- |
| **(Popping et al. 2019)** | MSM | Netherlands | Deterministic mathematical model | Early HCV treatment (3 scenarios) |
| **(Kahn et al. 2011)** | Not specified | International | Review | Expanded ART for prevention |
| **(Wilson and Fraser 2014)** | Not specified | International | Review | Treatment as Prevention |
| **(Nosyk et al. 2015)** | Not specified | Canada | Dynamic, compartmental transmission model | ART scale-up compared with hypothetical scenarios of constrained treatment access. |
| **(Ogbuagu and Bruce 2014)** | Different high risk groups | International | Review | Treatment as Prevention |
| **(Venkatesh et al. 2010)** | PLWH | International | Mathematical modelling | Expanded HIV treatment |
| **(Johnston et al. 2010)** | PLWH | Canada | Mathematical model describing transmission | Expanding access to HIV Treatment (HAART) |
| **(Pinkerton et al. 2013)** | PLWH | USA | Mathematical modelling | US AIDS Drug Assistance Program which provides access to prescription drugs |
| **(Forsythe et al. 2019)** | PLWH | International | Modeling Approach (Spectrum package of models) | Improvements in HIV treatment coverage and effectiveness |

| **Combination: Test & Treat (5)** |
| --- |

| **(Nosyk et al. 2014)** | Different target groups | International | Review | Interventions to improve the cascade of HIV care |
| --- | --- | --- | --- | --- |
| **(Nosyk et al. 2018)** | PLWH | Canada | Dynamic HIV transmission model | Primary care testing, ART initiation, and ART retention initiatives |
| **(Long et al. 2010)** | (A) High-risk (IDUs, MSM)  (B) low-risk individuals | USA | Dynamic mathematical model of HIV transmission and disease progression, and cost-effectiveness analysis | Expanded HIV screening, counselling, treatment with ART |
| **(Lazenby et al. 2014)** | HIV-Positive Women | USA | A decision tree analysis | Annual Trichomonas screening and treatment |
| **(Chahal et al. 2019)** | Different high risk groups | USA | A lifetime Markov model | Hepatitis B screening and treatment or vaccination in high-risk populations |

| **Vaccination (6)** |
| --- |

| **(Jacobs and Meyerhoff 2003)** | Adults at public STD clinics | USA | Markov model | Substituting hepatitis A/B vaccine versus B vaccination |
| --- | --- | --- | --- | --- |
| **(Krahn et al. 2005)** | IDUs & all 12 year olds | Canada | Markov cohort simulation model | Universal vaccination with a hepatitis C vaccine (compared to no vaccination) |
| **(Desai et al. 2008)** | MSM | USA | Stochastic compartmental mathematical model | HIV chemoprophylaxis |
| **(Gray et al. 2011)** | MSM | Australia | individual-based stochastic computer simulation model | (Partially effective) HIV vaccine |
| **(Long and Owens 2011)** | General population & high risk populations | USA | Mathematic modelling | Different HIV vaccine scenarios |
| **(Owusu-Edusei et al. 2015)** | Young women | USA | Compartmental heterosexual transmission model. | Chlamydia Vaccination |

| **Pre-exposure prophylaxis (PrEP) (23)** |
| --- |

| **Reference** | **Target group(s) / population** | **Country** | **Design / Model reported** | **Intervention(s)** |
| --- | --- | --- | --- | --- |
| **(Schackman and Eggman 2012)** | Different target groups | International | Review | PrEP |
| **(Gomez et al. 2013)** | Different target groups | International | Review | PrEP |
| **(Hellinger 2013)** | Different target groups | International | Review | PrEP |
| **(Cambiano et al. 2016)** | Different target groups | International | Review | PrEP |
| **(Paltiel et al. 2009)** | MSM | USA | Individual-level state transition Monte Carlo simulation model | PrEP |
| **(Juusola et al. 2012)** | MSM | USA | Risk-based dynamic compartmental model of transmission | PrEP for the general MSM  population and PrEP for high-risk MSM |
| **(Chen and Dowdy 2014)** | MSM | USA | Decision-analytic model (with different scenarios) | PrEP |
| **(Drabo et al. 2016)** | MSM | USA | Mathematical epidemiological model | 12 different strategies of PrEP and Test & Treat (T&T) |
| **(McKenney et al. 2017)** | MSM | USA | Decision-analysis model with different scenarios | PrEP |
| **(Adamson et al. 2017)** | MSM | USA | Markov model based on clinical trial data | Comparison of standard HIV prevention, daily PrEP, HIV vaccine, & a combination |
| **(Shen et al. 2018)** | MSM | USA | Infection–age-structured mathematical model | PrEP coverage with earlier ART |
| **(Schneider et al. 2014)** | MSM | Australia | Modelling analysis (stochastic agent-based model), Microcosting methods | PrEP |
| **(Ouellet et al. 2015)** | MSM | Australia | Economic evaluation (cost benefit) | On demand PrEP |
| **(Nichols et al. 2016)** | MSM | Netherlands | Deterministic mathematical model | Daily vs. on-demand PrEP |
| **(Durand-Zaleski et al. 2018)** | MSM | France | Prospective economic evaluation | PrEP |
| **(Cambiano et al. 2018)** | MSM | UK | Dynamic, individual-based stochastic model | Event-based PrEP program |
| **(van de Vijver et al. 2019)** | MSM | Germany | Deterministic mathematical model | PrEP |
| **(Mitchell et al. 2018)** | MSM & heterosexual populations | International | Systematic review and data triangulation for estimations | Non-daily versus daily oral PrEP |
| **(Bernard et al. 2016)** | IDUs | USA | Calibrated dynamic compartmental model | Different scenarios: (1) PrEP alone, (2) PrEP+screen, (3) PrEP+screen+ART.  All |
| **(Fu et al. 2018)** | IDUs | USA | Dynamic network model | Different strategies: (1) random IDUs (2) randomly selected and with partners (3) with most sexual and needle-sharing partners (4) with most positive partners |
| **(Letchumanan et al. 2015)** | Heterosexual serodiscordant couples (where the male  partner is HIV-positive) | Canada | A cohort Markov model | (1) condomless sex restricted to timed ovulation (CS) (2) condomless sex restricted to timed ovulation combined with PrEP (3) sperm washing with intrauterine insemination |
| **(Leech et al. 2018)** | Serodiscordant couples seeking contraception | USA | Markov cohort simulation model | PrEP compared with alternative  strategies |
| **(Khurana et al. 2018)** | (Khurana et al. 2018)Sexually active population / different subpopulations | USA | Dynamic, compartmental model | PrEP (when improving diagnosis, care, and treatment of PLWH) |

| **Postexposure prophylaxis (PEP) (2)** |
| --- |

| **(Pinkerton et al. 2004)** | Different target groups | USA | Retrospective cost analysis combined with model-based effectiveness estimates to determine “cost-utility ratio” | PEP for non-occupational HIV exposures (Intervention after sexual or injection-drug use exposure) |
| --- | --- | --- | --- | --- |
| **(Herida et al. 2006)** | Different target groups | France | Cost-effectiveness analysis based on a decision tree | PEP |

| **Mother-to-child transmission (MTCT) (12)** |
| --- |

| **Reference** | **Target group(s) / population** | **Country** | **Design / Model reported** | **Intervention(s)** |
| --- | --- | --- | --- | --- |
| **(Ruger et al. 2014)** | Pregnant women | International | Review | PMTCT (drug abuse treatment and HIV prevention programs) |
| **(Bert et al. 2018)** | Pregnant women | International | Review | Antenatal screening strategy to prevent vertical transmission |
| **(Graves et al. 2004)** | Pregnant women | Australia | Mathematic modelling | Universal antenatal HIV screening |
| **(Ong et al. 2016)** | Pregnant women | Australia | Cost effectiveness based on a decision model | (1) HIV Screening all pregnant women aged 16–25 years for chlamydia compared with (2) Selective screening or (3) no screening. |
| **(Sansom et al. 2003)** | Pregnant women | USA | Decision tree in cost-effectiveness analysis | (1) high-risk communities (2) nationwide HIV testing strategies |
| **(Mrus and Tsevat 2004)** | Pregnant women | USA | Decision analytic model | Offering rapid HIV testing and treatment to women without prenatal care testing positive compared with no testing |
| **(Resch et al. 2005)** | Pregnant women | USA | Decision analytic model | (1) Voluntary prenatal screening (2) Routine prenatal screening [RPS], (3) mandatory newborn screening for a high-risk population of incarcerated pregnant women. |
| **(Ditkowsky et al. 2017)** | Pregnant women | USA | Decision analysis model | Screening pregnant young women for C. trachomatis in a high burden setting |
| **(Hersh et al. 2018)** | Pregnant women | USA | Cost-effectiveness model | Screening for Syphilis |
| **(Halpern et al. 2000)** | HIV infected pregnant women | USA | Random-mixing epidemiological model & steady-state analysis | Elective caesarean section vs. vaginal delivery |
| **(Mrus et al. 2000)** | HIV-infected pregnant women | USA | Cost-effectiveness analysis using a probabilistic decision model. | Elective caesarean section vs. vaginal delivery |
| **(McCabe et al. 2010)** | HIV-infected pregnant women | USA | Mathematical model, Monte Carlo simulations | (1) self-administered (2) directly observed antiretroviral therapy (3) no HAART |

| **Comparisons and combinations of different interventions (11)** |
| --- |

| **(Jacobsen and Walensky 2016)** | Different target groups | International | Review | Condoms and circumcision, behavioural or community-based interventions, PMTCT, HIV testing, PrEP, TasP |
| --- | --- | --- | --- | --- |
| **(Cohen et al. 2004)** | Different target groups | USA | Bernoulli and proportionate change model | 26 HIV prevention interventions including biomedical interventions, structural interventions, and interventions designed to change risk behaviours of individuals |
| **(Huang et al. 2015)** | Different target groups | USA | Review | HIV testing, prevention with HIV-positives and their partners, condom distribution, policy initiatives |
| **(Lin et al. 2016)** | Different target groups | USA | Bernoulli process model | HIV testing and partner services; care- and treatment-related interventions; behavioural interventions; PrEP; adult circumcision |
| **(Barham et al. 2007)** | Youth | International | Review | Different one to one interventions |
| **(Brent et al. 2010)** | PLWH | International | Review | HIV prevention with focus on the aging of the HIV-positive population |
| **(Holtgrave et al. 2012a)** | PLWH | USA | Economic evaluation and epidemiologic modeling | Different policy scenarios |
| **(John-Baptiste et al. 2012)** | Different target groups (i.a. IDUs) | International | Review | HCV interventions (prevention, screening, treatment) |
| **(Selvapatt et al. 2017)** | IDUs | UK | Cost-effective-analysis using Markov model | Outreach testing and treatment for HCV in an urban Drug Treatment Unit. |
| **(Bernard et al. 2017)** | IDUs | USA | Empirically calibrated dynamic compartmental model | Combinations of: opioid agonist therapy, needle and syringe programs, HIV testing and treatment, and oral HIV pre-exposure prophylaxis |
| **(Tuli and Kerndt 2009)** | MSM (imprisoned) | USA | Mathematical model | Screening, treatment, and condom provision intervention |

References

Adams, Elisabeth J.; Turner, Katherine M. E.; Edmunds, W. John (2007): The cost effectiveness of opportunistic chlamydia screening in England. In *Sexually transmitted infections* 83 (4), 267-74; discussion 274-5. DOI: 10.1136/sti.2006.024364.

Adamson, Blythe J. S.; Carlson, Josh J.; Kublin, James G.; Garrison, Louis P. (2017): The Potential Cost-Effectiveness of Pre-Exposure Prophylaxis Combined with HIV Vaccines in the United States. In *Vaccines* 5 (2). DOI: 10.3390/vaccines5020013.

Aledort, Julia E.; Hook, Edward W.; Weinstein, Milton C.; Goldie, Sue J. (2005): The cost effectiveness of gonorrhea screening in urban emergency departments. In *Sexually transmitted diseases* 32 (7), pp. 425–436. DOI: 10.1097/01.olq.0000154501.22566.fa.

Baggaley, Rebecca F.; Irvine, Michael A.; Leber, Werner; Cambiano, Valentina; Figueroa, Jose; McMullen, Heather et al. (2017): Cost-effectiveness of screening for HIV in primary care: a health economics modelling analysis. In *The Lancet HIV* 4 (10), e465-e474. DOI: 10.1016/S2352-3018(17)30123-6.

Barham, L.; Lewis, D.; Latimer, N. (2007): One to one interventions to reduce sexually transmitted infections and under the age of 18 conceptions: a systematic review of the economic evaluations. In *Sexually transmitted infections* 83 (6), pp. 441–446. DOI: 10.1136/sti.2007.025361.

Bedimo, Ariane Lisann; Pinkerton, Steven D.; Cohen, Deborah A.; Gray, Bradley; Farley, Thomas A. (2002): Condom distribution: a cost-utility analysis. In *International journal of STD & AIDS* 13 (6), pp. 384–392. DOI: 10.1258/095646202760029804.

Bernard, Cora L.; Brandeau, Margaret L.; Humphreys, Keith; Bendavid, Eran; Holodniy, Mark; Weyant, Christopher et al. (2016): Cost-Effectiveness of HIV Preexposure Prophylaxis for People Who Inject Drugs in the United States. In *Annals of internal medicine*. DOI: 10.7326/M15-2634.

Bernard, Cora L.; Owens, Douglas K.; Goldhaber-Fiebert, Jeremy D.; Brandeau, Margaret L. (2017): Estimation of the cost-effectiveness of HIV prevention portfolios for people who inject drugs in the United States: A model-based analysis. In *PLoS medicine* 14 (5), e1002312. DOI: 10.1371/journal.pmed.1002312.

Bert, Fabrizio; Gualano, Maria Rosaria; Biancone, Paolo; Brescia, Valerio; Camussi, Elisa; Martorana, Maria et al. (2018): HIV screening in pregnant women: A systematic review of cost-effectiveness studies. In *The International journal of health planning and management* 33 (1), pp. 31–50. DOI: 10.1002/hpm.2418.

Bos, J. M.; Fennema, J. S.; Postma, M. J. (2001): Cost-effectiveness of HIV screening of patients attending clinics for sexually transmitted diseases in Amsterdam. In *AIDS (London, England)* 15 (15), pp. 2031–2036. DOI: 10.1097/00002030-200110190-00017.

Brent, Robert J.; Brennan, Mark; Karpiak, Stephen E. (2010): Economic evaluations of HIV prevention in rich countries and the need to focus on the aging of the HIV-positive population. In *Current opinion in HIV and AIDS* 5 (3), pp. 255–260. DOI: 10.1097/COH.0b013e3283384a88.

Burgos, José L.; Gaebler, Julia A.; Strathdee, Steffanie A.; Lozada, Remedios; Staines, Hugo; Patterson, Thomas L. (2010): Cost-Effectiveness of an Intervention to Reduce HIV/STI Incidence and Promote Condom Use among Female Sex Workers in the Mexico–US Border Region. In *PloS one* 5 (6). DOI: 10.1371/journal.pone.0011413.

Cambiano, Valentina; Miners, Alec; Dunn, David; McCormack, Sheena; Ong, Koh Jun; Gill, O. Noel et al. (2018): Cost-effectiveness of pre-exposure prophylaxis for HIV prevention in men who have sex with men in the UK: a modelling study and health economic evaluation. In *The Lancet Infectious Diseases* 18 (1), pp. 85–94. DOI: 10.1016/S1473-3099(17)30540-6.

Cambiano, Valentina; Miners, Alec; Phillips, Andrew (2016): What do we know about the cost-effectiveness of HIV preexposure prophylaxis, and is it affordable? In *Current opinion in HIV and AIDS* 11 (1), pp. 56–66. DOI: 10.1097/COH.0000000000000217.

Castel, Amanda D.; Choi, Sungwoog; Dor, Avi; Skillicorn, Jennifer; Peterson, James; Rocha, Nestor; Kharfen, Michael (2015): Comparing Cost-Effectiveness of HIV Testing Strategies: Targeted and Routine Testing in Washington, DC. In *PloS one* 10 (10), e0139605. DOI: 10.1371/journal.pone.0139605.

Chahal, Harinder S.; Peters, Marion G.; Harris, Aaron M.; McCabe, Devon; Volberding, Paul; Kahn, James G. (2019): Cost-effectiveness of Hepatitis B Virus Infection Screening and Treatment or Vaccination in 6 High-risk Populations in the United States. In *Open forum infectious diseases* 6 (1), ofy353. DOI: 10.1093/ofid/ofy353.

Chen, Anders; Dowdy, David W. (2014): Clinical effectiveness and cost-effectiveness of HIV pre-exposure prophylaxis in men who have sex with men: risk calculators for real-world decision-making. In *PloS one* 9 (10), e108742. DOI: 10.1371/journal.pone.0108742.

Chesson, Harrell W. (2006): Estimated effectiveness and cost-effectiveness of federally funded prevention efforts on gonorrhea rates in the United States, 1971-2003, under various assumptions about the impact of prevention funding. In *Sexually transmitted diseases* 33 (10 Suppl), S140-4. DOI: 10.1097/01.olq.0000194575.79728.72.

Chesson, Harrell W. (2007): Cost effectiveness of one to one STI prevention interventions. In *Sexually transmitted infections* 83 (6), pp. 423–424. DOI: 10.1136/sti.2007.026641.

Chesson, Harrell W.; Bernstein, Kyle T.; Gift, Thomas L.; Marcus, Julia L.; Pipkin, Sharon; Kent, Charlotte K. (2013): The cost-effectiveness of screening men who have sex with men for rectal chlamydial and gonococcal infection to prevent HIV Infection. In *Sexually transmitted diseases* 40 (5), pp. 366–371. DOI: 10.1097/OLQ.0b013e318284e544.

Chesson, Harrell W.; Kidd, Sarah; Bernstein, Kyle T.; Fanfair, Robyn Neblett; Gift, Thomas L. (2016): The Cost-Effectiveness of Syphilis Screening Among Men Who Have Sex With Men: An Exploratory Modeling Analysis. In *Sexually transmitted diseases* 43 (7), pp. 429–432. DOI: 10.1097/OLQ.0000000000000461.

Choi, Stephanie K. Y.; Holtgrave, David R.; Bacon, Jean; Kennedy, Rick; Lush, Joanne; McGee, Frank et al. (2016): Economic Evaluation of Community-Based HIV Prevention Programs in Ontario: Evidence of Effectiveness in Reducing HIV Infections and Health Care Costs. In *AIDS and behavior* 20 (6), pp. 1143–1156. DOI: 10.1007/s10461-015-1109-8.

Cohen, Deborah A.; Wu, Shin-Yi; Farley, Thomas A. (2004): Comparing the cost-effectiveness of HIV prevention interventions. In *Journal of acquired immune deficiency syndromes (1999)* 37 (3), pp. 1404–1414. DOI: 10.1097/01.qai.0000123271.76723.96.

Cohen, Deborah A.; Wu, Shin-Yi; Farley, Thomas A. (2006): Structural interventions to prevent HIV/sexually transmitted disease: are they cost-effective for women in the southern United States? In *Sexually transmitted diseases* 33 (7 Suppl), S46-9. DOI: 10.1097/01.olq.0000221015.64056.ee.

Cooper, Keith; Shepherd, Jonathan; Picot, Jo; Jones, Jeremy; Kavanagh, Josephine; Harden, Angela et al. (2012): An economic model of school-based behavioral interventions to prevent sexually transmitted infections. In *International journal of technology assessment in health care* 28 (4), pp. 407–414. DOI: 10.1017/S0266462312000475.

Dauner, Kim Nichols; Oglesby, Willie H.; Richter, Donna L.; LaRose, Christopher M.; Holtgrave, David R. (2008): Cost savings threshold analysis of a capacity-building program for HIV prevention organizations. In *AIDS education and prevention : official publication of the International Society for AIDS Education* 20 (3), pp. 265–274. DOI: 10.1521/aeap.2008.20.3.265.

Deogan, Charlotte L.; Bocangel, Marta K. Hansson; Wamala, Sarah P.; Månsdotter, Anna M. (2010): A cost-effectiveness analysis of the Chlamydia Monday--a community-based intervention to decrease the prevalence of chlamydia in Sweden. In *Scandinavian journal of public health* 38 (2), pp. 141–150. DOI: 10.1177/1403494809357260.

Desai, Kamal; Sansom, Stephanie L.; Ackers, Marta L.; Stewart, Scott R.; Hall, H. Irene; Hu, Dale J. et al. (2008): Modeling the impact of HIV chemoprophylaxis strategies among men who have sex with men in the United States: HIV infections prevented and cost-effectiveness. In *AIDS (London, England)* 22 (14), pp. 1829–1839. DOI: 10.1097/QAD.0b013e32830e00f5.

Deuffic-Burban, Sylvie; Huneau, Alexandre; Verleene, Adeline; Brouard, Cécile; Pillonel, Josiane; Le Strat, Yann et al. (2018): Assessing the cost-effectiveness of hepatitis C screening strategies in France. In *Journal of hepatology* 69 (4), pp. 785–792. DOI: 10.1016/j.jhep.2018.05.027.

Ditkowsky, Jared; Shah, Khushal H.; Hammerschlag, Margaret R.; Kohlhoff, Stephan; Smith-Norowitz, Tamar A. (2017): Cost-benefit analysis of Chlamydia trachomatis screening in pregnant women in a high burden setting in the United States. In *BMC infectious diseases* 17 (1), p. 155. DOI: 10.1186/s12879-017-2248-5.

Dowdy, David W.; Rodriguez, Robert M.; Hare, C. Bradley; Kaplan, Beth (2011): Cost-effectiveness of targeted human immunodeficiency virus screening in an urban emergency department. In *Academic emergency medicine : official journal of the Society for Academic Emergency Medicine* 18 (7), pp. 745–753. DOI: 10.1111/j.1553-2712.2011.01110.x.

Drabo, Emmanuel F.; Hay, Joel W.; Vardavas, Raffaele; Wagner, Zachary R.; Sood, Neeraj (2016): A Cost-effectiveness Analysis of Preexposure Prophylaxis for the Prevention of HIV Among Los Angeles County Men Who Have Sex With Men. In *Clinical infectious diseases : an official publication of the Infectious Diseases Society of America* 63 (11), pp. 1495–1504. DOI: 10.1093/cid/ciw578.

Durand-Zaleski, Isabelle; Mutuon, Pierre; Charreau, Isabelle; Tremblay, Cecile; Rojas, Daniela; Pialoux, Gilles et al. (2018): Costs and benefits of on-demand HIV preexposure prophylaxis in MSM. In *AIDS (London, England)* 32 (1), pp. 95–102. DOI: 10.1097/QAD.0000000000001658.

Forsythe, Steven S.; McGreevey, William; Whiteside, Alan; Shah, Maunank; Cohen, Joshua; Hecht, Robert et al. (2019): Twenty Years Of Antiretroviral Therapy For People Living With HIV: Global Costs, Health Achievements, Economic Benefits. In *Health affairs (Project Hope)* 38 (7), pp. 1163–1172. DOI: 10.1377/hlthaff.2018.05391.

Fu, Rui; Owens, Douglas K.; Brandeau, Margaret L. (2018): Cost-effectiveness of alternative strategies for provision of HIV preexposure prophylaxis for people who inject drugs. In *AIDS (London, England)* 32 (5), pp. 663–672. DOI: 10.1097/QAD.0000000000001747.

Gift, Thomas L.; Gaydos, Charlotte A.; Kent, Charlotte K.; Marrazzo, Jeanne M.; Rietmeijer, Cornelis A.; Schillinger, Julia A.; Dunne, Eileen F. (2008): The program cost and cost-effectiveness of screening men for Chlamydia to prevent pelvic inflammatory disease in women. In *Sexually transmitted diseases* 35 (11 Suppl), S66-75. DOI: 10.1097/OLQ.0b013e31818b64ac.

Gillespie, Paddy; O'Neill, Ciaran; Adams, Elisabeth; Turner, Katherine; O'Donovan, Diarmuid; Brugha, Ruairi et al. (2012): The cost and cost-effectiveness of opportunistic screening for Chlamydia trachomatis in Ireland. In *Sexually transmitted infections* 88 (3), pp. 222–228. DOI: 10.1136/sextrans-2011-050067.

Gomez, Gabriela B.; Borquez, Annick; Case, Kelsey K.; Wheelock, Ana; Vassall, Anna; Hankins, Catherine (2013): The cost and impact of scaling up pre-exposure prophylaxis for HIV prevention: a systematic review of cost-effectiveness modelling studies. In *PLoS medicine* 10 (3), e1001401. DOI: 10.1371/journal.pmed.1001401.

Gopalappa, Chaitra; Farnham, Paul G.; Hutchinson, Angela B.; Sansom, Stephanie L. (2012): Cost effectiveness of the National HIV/AIDS Strategy goal of increasing linkage to care for HIV-infected persons. In *Journal of acquired immune deficiency syndromes (1999)* 61 (1), pp. 99–105. DOI: 10.1097/QAI.0b013e31825bd862.

Graves, Nicholas; Walker, Damian G.; McDonald, Ann M.; Kaldor, John M.; Ziegler, John B. (2004): Would universal antenatal screening for HIV infection be cost-effective in a setting of very low prevalence? Modelling the data for Australia. In *The Journal of infectious diseases* 190 (1), pp. 166–174. DOI: 10.1086/421247.

Gray, Richard T.; Ghaus, Mohammad H.; Hoare, Alexander; Wilson, David P. (2011): Expected epidemiological impact of the introduction of a partially effective HIV vaccine among men who have sex with men in Australia. In *Vaccine* 29 (36), pp. 6125–6129. DOI: 10.1016/j.vaccine.2011.06.061.

Halpern, M. T.; Read, J. S.; Ganoczy, D. A.; Harris, D. R. (2000): Cost-effectiveness of cesarean section delivery to prevent mother-to-child transmission of HIV-1. In *AIDS (London, England)* 14 (6), pp. 691–700. DOI: 10.1097/00002030-200004140-00008.

Harte, Derval; Mercey, Danielle; Jarman, Jay; Benn, Paul (2011): Is the recall of men who have sex with men (MSM) diagnosed as having bacterial sexually transmitted infections (STIs) for re-screening a feasible and effective strategy? In *Sexually transmitted infections* 87 (7), pp. 577–582. DOI: 10.1136/sextrans-2011-050144.

Haukoos, Jason S.; Campbell, Jonathan D.; Conroy, Amy A.; Hopkins, Emily; Bucossi, Meggan M.; Sasson, Comilla et al. (2013): Programmatic cost evaluation of nontargeted opt-out rapid HIV screening in the emergency department. In *PloS one* 8 (12), e81565. DOI: 10.1371/journal.pone.0081565.

Hellinger, Fred J. (2013): Assessing the cost effectiveness of pre-exposure prophylaxis for HIV prevention in the US. In *PharmacoEconomics* 31 (12), pp. 1091–1104. DOI: 10.1007/s40273-013-0111-0.

Helsper, C. W.; Borkent-Raven, B. A.; Wit, N. J. de; van Essen, G. A.; Bonten, M. J. M.; Hoepelman, A. I. M. et al. (2012): Cost-effectiveness of targeted screening for hepatitis C in The Netherlands. In *Epidemiology and infection* 140 (1), pp. 58–69. DOI: 10.1017/S0950268811000112.

Herbst, Jeffrey H.; Beeker, Carolyn; Mathew, Anita; McNally, Tarra; Passin, Warren F.; Kay, Linda S. et al. (2007): The effectiveness of individual-, group-, and community-level HIV behavioral risk-reduction interventions for adult men who have sex with men: a systematic review. In *American journal of preventive medicine* 32 (4 Suppl), S38-67. DOI: 10.1016/j.amepre.2006.12.006.

Herida, Magid; Larsen, Christine; Lot, Florence; Laporte, Anne; Desenclos, Jean-Claude; Hamers, Françoise F. (2006): Cost-effectiveness of HIV post-exposure prophylaxis in France. In *AIDS (London, England)* 20 (13), pp. 1753–1761. DOI: 10.1097/01.aids.0000242822.74624.5f.

Hersh, Alyssa R.; Megli, Christina J.; Caughey, Aaron B. (2018): Repeat Screening for Syphilis in the Third Trimester of Pregnancy: A Cost-Effectiveness Analysis. In *Obstetrics and gynecology* 132 (3), pp. 699–707. DOI: 10.1097/AOG.0000000000002795.

Heumann, K. S.; Marx, R.; Lawrence, S. J.; Stump, D. L.; Carroll, D. P.; Hirozawa, A. M. et al. (2001): Cost-effectiveness of prevention referrals for high-risk HIV-negatives in San Francisco. In *AIDS care* 13 (5), pp. 637–642. DOI: 10.1080/09540120120063269.

Hoenigl, Martin; Chaillon, Antoine; Mehta, Sanjay R.; Smith, Davey M.; Graff-Zivin, Joshua; Little, Susan J. (2016): Screening for acute HIV infection in community-based settings: Cost-effectiveness and impact on transmissions. In *The Journal of infection* 73 (5), pp. 476–484. DOI: 10.1016/j.jinf.2016.07.019.

Holtgrave, David R. (2007a): Costs and consequences of the US Centers for Disease Control and Prevention's recommendations for opt-out HIV testing. In *PLoS medicine* 4 (6), e194. DOI: 10.1371/journal.pmed.0040194.

Holtgrave, David R. (2007b): The president's fiscal year 2007 initiative for human immunodeficiency virus counseling and testing expansion in the United States: a scenario analysis of its coverage, impact, and cost-effectiveness. In *Journal of public health management and practice : JPHMP* 13 (3), pp. 239–243. DOI: 10.1097/01.PHH.0000267681.00659.e5.

Holtgrave, David R.; Hall, H. Irene; Wehrmeyer, Laura; Maulsby, Cathy (2012a): Costs, consequences and feasibility of strategies for achieving the goals of the National HIV/AIDS strategy in the United States: a closing window for success? In *AIDS and behavior* 16 (6), pp. 1365–1372. DOI: 10.1007/s10461-012-0207-0.

Holtgrave, David R.; Maulsby, Catherine; Kharfen, Michael; Jia, Yujiang; Wu, Charles; Opoku, Jenevieve et al. (2012b): Cost-utility analysis of a female condom promotion program in Washington, DC. In *AIDS and behavior* 16 (5), pp. 1115–1120. DOI: 10.1007/s10461-012-0174-5.

Hsu, Justine; Zinsou, Cyprien; Parkhurst, Justin; N'Dour, Marguerite; Foyet, Léger; Mueller, Dirk H. (2013): Comparative costs and cost-effectiveness of behavioural interventions as part of HIV prevention strategies. In *Health policy and planning* 28 (1), pp. 20–29. DOI: 10.1093/heapol/czs021.

Hu, Delphine; Hook, Edward W.; Goldie, Sue J. (2004): Screening for Chlamydia trachomatis in women 15 to 29 years of age: a cost-effectiveness analysis. In *Annals of internal medicine* 141 (7), pp. 501–513. DOI: 10.7326/0003-4819-141-7-200410050-00006.

Huang, Ya-Lin A.; Lasry, Arielle; Hutchinson, Angela B.; Sansom, Stephanie L. (2015): A systematic review on cost effectiveness of HIV prevention interventions in the United States. In *Applied health economics and health policy* 13 (2), pp. 149–156. DOI: 10.1007/s40258-014-0142-5.

Hutchinson, Angela B.; Farnham, Paul G.; Sansom, Stephanie L.; Yaylali, Emine; Mermin, Jonathan H. (2016): Cost-Effectiveness of Frequent HIV Testing of High-Risk Populations in the United States. In *Journal of acquired immune deficiency syndromes (1999)* 71 (3), pp. 323–330. DOI: 10.1097/QAI.0000000000000838.

Jacobs, R. Jake; Meyerhoff, Allen S. (2003): Cost-effectiveness of hepatitis A/B vaccine versus hepatitis B vaccine in public sexually transmitted disease clinics. In *Sexually transmitted diseases* 30 (11), pp. 859–865. DOI: 10.1097/01.OLQ.0000086601.18907.47.

Jacobsen, Margo M.; Walensky, Rochelle P. (2016): Modeling and Cost-Effectiveness in HIV Prevention. In *Current HIV/AIDS reports* 13 (1), pp. 64–75. DOI: 10.1007/s11904-016-0303-2.

Jain, Kriti M.; Maulsby, Catherine; Brantley, Meredith; Kim, Jeeyon Janet; Zulliger, Rose; Riordan, Maura et al. (2016): Cost and cost threshold analyses for 12 innovative US HIV linkage and retention in care programs. In *AIDS care* 28 (9), pp. 1199–1204. DOI: 10.1080/09540121.2016.1164294.

John-Baptiste, Ava; Yeung, Man Wah; Leung, Victoria; van der Velde, Gabrielle; Krahn, Murray (2012): Cost effectiveness of hepatitis C-related interventions targeting substance users and other high-risk groups: a systematic review. In *PharmacoEconomics* 30 (11), pp. 1015–1034. DOI: 10.2165/11597660-000000000-00000.

Johnson, A. P.; Macgowan, R. J.; Eldridge, G. D.; Morrow, K. M.; Sosman, J.; Zack, B.; Margolis, A. (2013): Cost and threshold analysis of an HIV/STI/hepatitis prevention intervention for young men leaving prison: Project START. In *AIDS and behavior* 17 (8), pp. 2676–2684. DOI: 10.1007/s10461-011-0096-7.

Johnson-Masotti, A. P.; Pinkerton, S. D.; Kelly, J. A.; Stevenson, L. Y. (2000): Cost-effectiveness of an HIV risk reduction intervention for adults with severe mental illness. In *AIDS care* 12 (3), pp. 321–332. DOI: 10.1080/09540120050042981.

Johnson-Masotti, Ana P.; Pinkerton, Steven D.; Sikkema, Kathleen J.; Kelly, Jeffrey A.; Wagstaff, David A. (2005): Cost-effectiveness of a community-level HIV risk reduction intervention for women living in low-income housing developments. In *The journal of primary prevention* 26 (4), pp. 345–362. DOI: 10.1007/s10935-005-5392-2.

Johnston, Karissa M.; Levy, Adrian R.; Lima, Viviane D.; Hogg, Robert S.; Tyndall, Mark W.; Gustafson, Paul et al. (2010): Expanding access to HAART: a cost-effective approach for treating and preventing HIV. In *AIDS (London, England)* 24 (12), pp. 1929–1935. DOI: 10.1097/QAD.0b013e32833af85d.

Juusola, Jessie L.; Brandeau, Margaret L.; Long, Elisa F.; Owens, Douglas K.; Bendavid, Eran (2011): The cost-effectiveness of symptom-based testing and routine screening for acute HIV infection in men who have sex with men in the USA. In *AIDS (London, England)* 25 (14), pp. 1779–1787. DOI: 10.1097/QAD.0b013e328349f067.

Juusola, Jessie L.; Brandeau, Margaret L.; Owens, Douglas K.; Bendavid, Eran (2012): The Cost-Effectiveness of Preexposure Prophylaxis for HIV Prevention in Men Who Have Sex with Men in the United States. In *Annals of internal medicine* 156 (8), pp. 541–550. DOI: 10.1059/0003-4819-156-8-201204170-00001.

Kahn, J. G.; Kegeles, S. M.; Hays, R.; Beltzer, N. (2001): Cost-effectiveness of the Mpowerment Project, a community-level intervention for young gay men. In *Journal of acquired immune deficiency syndromes (1999)* 27 (5), pp. 482–491. DOI: 10.1097/00126334-200108150-00010.

Kahn, James G.; Marseille, Elliot A.; Bennett, Rod; Williams, Brian G.; Granich, Reuben (2011): Cost-effectiveness of antiretroviral therapy for prevention. In *Current HIV research* 9 (6), pp. 405–415. DOI: 10.2174/157016211798038542.

Khurana, Nidhi; Yaylali, Emine; Farnham, Paul G.; Hicks, Katherine A.; Allaire, Benjamin T.; Jacobson, Evin; Sansom, Stephanie L. (2018): Impact of Improved HIV Care and Treatment on PrEP Effectiveness in the United States, 2016-2020. In *Journal of acquired immune deficiency syndromes (1999)* 78 (4), pp. 399–405. DOI: 10.1097/QAI.0000000000001707.

Krahn, Murray D.; John-Baptiste, Ava; Yi, Qilong; Doria, Andrea; Remis, Robert S.; Ritvo, Paul; Friedman, Samuel (2005): Potential cost-effectiveness of a preventive hepatitis C vaccine in high risk and average risk populations in Canada. In *Vaccine* 23 (13), pp. 1549–1558. DOI: 10.1016/j.vaccine.2004.09.023.

Kwon, Jisoo A.; Anderson, Jonathan; Kerr, Cliff C.; Thein, Hla-Hla; Zhang, Lei; Iversen, Jenny et al. (2012): Estimating the cost-effectiveness of needle-syringe programs in Australia. In *AIDS (London, England)* 26 (17), pp. 2201–2210. DOI: 10.1097/QAD.0b013e3283578b5d.

Lazenby, Gweneth Bratton; Unal, Elizabeth Ramsey; Andrews, Annie Lintzenich; Simpson, Kit (2014): Cost-effectiveness analysis of annual Trichomonas vaginalis screening and treatment in HIV-positive women to prevent HIV transmission. In *Sexually transmitted diseases* 41 (6), pp. 353–358. DOI: 10.1097/OLQ.0000000000000008.

Leech, Ashley A.; Burgess, James F.; Sullivan, Meg; Kuohung, Wendy; Horný, Michal; Drainoni, Mari-Lynn et al. (2018): Cost-effectiveness of preexposure prophylaxis for HIV prevention for conception in the United States. In *AIDS (London, England)* 32 (18), pp. 2787–2798. DOI: 10.1097/QAD.0000000000002014.

Letchumanan, Michelle; Coyte, Peter C.; Loutfy, Mona (2015): An economic evaluation of conception strategies for heterosexual serodiscordant couples where the male partner is HIV-positive. In *Antiviral therapy* 20 (6), pp. 613–621. DOI: 10.3851/IMP2956.

Li, Xinqi C.; Kusi, Lillian; Marak, Theodore; Bertrand, Thomas; Chan, Philip A.; Galárraga, Omar (2018): The Cost and Cost-utility of Three Public Health HIV Case-finding Strategies: Evidence from Rhode Island, 2012-2014. In *AIDS and behavior* 22 (11), pp. 3726–3733. DOI: 10.1007/s10461-017-1940-1.

Lin, Feng; Farnham, Paul G.; Shrestha, Ram K.; Mermin, Jonathan; Sansom, Stephanie L. (2016): Cost Effectiveness of HIV Prevention Interventions in the U.S. In *American journal of preventive medicine* 50 (6), pp. 699–708. DOI: 10.1016/j.amepre.2016.01.011.

Long, Elisa F. (2011): HIV screening via fourth-generation immunoassay or nucleic acid amplification test in the United States: a cost-effectiveness analysis. In *PloS one* 6 (11), e27625. DOI: 10.1371/journal.pone.0027625.

Long, Elisa F.; Brandeau, Margaret L.; Owens, Douglas K. (2010): The cost-effectiveness and population outcomes of expanded HIV screening and antiretroviral treatment in the United States. In *Annals of internal medicine* 153 (12), pp. 778–789. DOI: 10.7326/0003-4819-153-12-201012210-00004.

Long, Elisa F.; Mandalia, Roshni; Mandalia, Sundhiya; Alistar, Sabina S.; Beck, Eduard J.; Brandeau, Margaret L. (2014): Expanded HIV testing in low-prevalence, high-income countries: a cost-effectiveness analysis for the United Kingdom. In *PloS one* 9 (4), e95735. DOI: 10.1371/journal.pone.0095735.

Long, Elisa F.; Owens, Douglas K. (2011): The cost-effectiveness of a modestly effective HIV vaccine in the United States. In *Vaccine* 29 (36), pp. 6113–6124. DOI: 10.1016/j.vaccine.2011.04.013.

Looker, Katharine J.; Wallace, Lesley A.; Turner, Katherine M. E. (2015): Impact and cost-effectiveness of chlamydia testing in Scotland: a mathematical modelling study. In *Theoretical biology & medical modelling* 12, p. 2. DOI: 10.1186/1742-4682-12-2.

Lucas, Aaron; Armbruster, Benjamin (2013): The cost-effectiveness of expanded HIV screening in the United States. In *AIDS (London, England)* 27 (5), pp. 795–801. DOI: 10.1097/QAD.0b013e32835c54f9.

Marseille, Elliot; Shade, Starley B.; Myers, Janet; Morin, Steve (2011): The cost-effectiveness of HIV prevention interventions for HIV-infected patients seen in clinical settings. In *Journal of acquired immune deficiency syndromes (1999)* 56 (3), e87-94. DOI: 10.1097/QAI.0b013e318204123e.

Martin, Erika G.; Paltiel, A. David; Walensky, Rochelle P.; Schackman, Bruce R. (2010): Expanded HIV screening in the United States: what will it cost government discretionary and entitlement programs? A budget impact analysis. In *Value in health : the journal of the International Society for Pharmacoeconomics and Outcomes Research* 13 (8), pp. 893–902. DOI: 10.1111/j.1524-4733.2010.00763.x.

Martin, Natasha K.; Vickerman, Peter; Khakoo, Salim; Ghosh, Anjan; Ramsay, Mary; Hickman, M. et al. (2019): Chronic hepatitis B virus case-finding in UK populations born abroad in intermediate or high endemicity countries: an economic evaluation. In *BMJ open* 9 (6), e030183. DOI: 10.1136/bmjopen-2019-030183.

Maulsby, Catherine; Jain, Kriti M.; Weir, Brian W.; Enobun, Blessing; Werner, Melissa; Riordan, Morey; Holtgrave, David R. (2018): Cost-Utility of Access to Care, a National HIV Linkage, Re-engagement and Retention in Care Program. In *AIDS and behavior* 22 (11), pp. 3734–3741. DOI: 10.1007/s10461-017-2015-z.

McCabe, Caitlin J.; Goldie, Sue J.; Fisman, David N. (2010): The cost-effectiveness of directly observed highly-active antiretroviral therapy in the third trimester in HIV-infected pregnant women. In *PloS one* 5 (4), e10154. DOI: 10.1371/journal.pone.0010154.

McKenney, Jennie; Chen, Anders; Hoover, Karen W.; Kelly, Jane; Dowdy, David; Sharifi, Parastu et al. (2017): Optimal costs of HIV pre-exposure prophylaxis for men who have sex with men. In *PloS one* 12 (6), e0178170. DOI: 10.1371/journal.pone.0178170.

Mitchell, Kate M.; Dimitrov, Dobromir; Hughes, James P.; Xia, Fan; Donnell, Deborah; Amico, K. Rivet et al. (2018): In what circumstances could nondaily preexposure prophylaxis for HIV substantially reduce program costs? In *AIDS (London, England)* 32 (6), pp. 809–818. DOI: 10.1097/QAD.0000000000001766.

Mrus, J. M.; Goldie, S. J.; Weinstein, M. C.; Tsevat, J. (2000): The cost-effectiveness of elective Cesarean delivery for HIV-infected women with detectable HIV RNA during pregnancy. In *AIDS (London, England)* 14 (16), pp. 2543–2552. DOI: 10.1097/00002030-200011100-00017.

Mrus, Joseph M.; Tsevat, Joel (2004): Cost-effectiveness of interventions to reduce vertical HIV transmission from pregnant women who have not received prenatal care. In *Medical decision making : an international journal of the Society for Medical Decision Making* 24 (1), pp. 30–39. DOI: 10.1177/0272989X03261570.

Nichols, Brooke E.; Boucher, Charles A. B.; van der Valk, Marc; Rijnders, Bart J. A.; van de Vijver, David A. M. C. (2016): Cost-effectiveness analysis of pre-exposure prophylaxis for HIV-1 prevention in the Netherlands: a mathematical modelling study. In *The Lancet Infectious Diseases* 16 (12), pp. 1423–1429. DOI: 10.1016/S1473-3099(16)30311-5.

Nichols, Brooke E.; Götz, Hannelore M.; van Gorp, Eric C. M.; Verbon, Annelies; Rokx, Casper; Boucher, Charles A. B.; van de Vijver, David A. M. C. (2015): Partner Notification for Reduction of HIV-1 Transmission and Related Costs among Men Who Have Sex with Men: A Mathematical Modeling Study. In *PloS one* 10 (11), e0142576. DOI: 10.1371/journal.pone.0142576.

Nosyk, B.; Krebs, E.; Eyawo, O.; Min, J. E.; Barrios, R.; Montaner, J. S. G. (2014): Cost-effectiveness analysis along the continuum of HIV care: how can we optimize the effect of HIV treatment as prevention programs? In *Current HIV/AIDS reports* 11 (4), pp. 468–478. DOI: 10.1007/s11904-014-0227-7.

Nosyk, Bohdan; Min, Jeong E.; Krebs, Emanuel; Zang, Xiao; Compton, Miranda; Gustafson, Reka et al. (2018): The Cost-Effectiveness of Human Immunodeficiency Virus Testing and Treatment Engagement Initiatives in British Columbia, Canada: 2011-2013. In *Clinical infectious diseases : an official publication of the Infectious Diseases Society of America* 66 (5), pp. 765–777. DOI: 10.1093/cid/cix832.

Nosyk, Bohdan; Min, Jeong E.; Lima, Viviane D.; Hogg, Robert S.; Montaner, Julio S. G. (2015): Cost-effectiveness of population-level expansion of highly active antiretroviral treatment for HIV in British Columbia, Canada: a modelling study. In *The Lancet HIV* 2 (9), e393-e400. DOI: 10.1016/S2352-3018(15)00127-7.

Ogbuagu, Onyema; Bruce, R. Douglas (2014): Reaching the unreached: treatment as prevention as a workable strategy to mitigate HIV and its consequences in high-risk groups. In *Current HIV/AIDS reports* 11 (4), pp. 505–512. DOI: 10.1007/s11904-014-0238-4.

Ong, J. J.; Chen, M.; Hocking, J.; Fairley, C. K.; Carter, R.; Bulfone, L.; Hsueh, A. (2016): Chlamydia screening for pregnant women aged 16-25 years attending an antenatal service: a cost-effectiveness study. In *BJOG : an international journal of obstetrics and gynaecology* 123 (7), pp. 1194–1202. DOI: 10.1111/1471-0528.13567.

Ouellet, Estelle; Durand, Madeleine; Guertin, Jason R.; LeLorier, Jacques; Tremblay, Cécile L. (2015): Cost effectiveness of 'on demand' HIV pre-exposure prophylaxis for non-injection drug-using men who have sex with men in Canada. In *The Canadian journal of infectious diseases & medical microbiology = Journal canadien des maladies infectieuses et de la microbiologie medicale* 26 (1), pp. 23–29. DOI: 10.1155/2015/964512.

Owusu-Edusei, Kwame; Chesson, Harrell W.; Gift, Thomas L.; Brunham, Robert C.; Bolan, Gail (2015): Cost-effectiveness of Chlamydia vaccination programs for young women. In *Emerging infectious diseases* 21 (6), pp. 960–968. DOI: 10.3201/eid2106.141270.

Paltiel, A. David; Freedberg, Kenneth A.; Scott, Callie A.; Schackman, Bruce R.; Losina, Elena; Wang, Bingxia et al. (2009): HIV preexposure prophylaxis in the United States: impact on lifetime infection risk, clinical outcomes, and cost-effectiveness. In *Clinical infectious diseases : an official publication of the Infectious Diseases Society of America* 48 (6), pp. 806–815. DOI: 10.1086/597095.

Paltiel, A. David; Walensky, Rochelle P.; Schackman, Bruce R.; Seage, George R.; Mercincavage, Lauren M.; Weinstein, Milton C.; Freedberg, Kenneth A. (2006): Expanded HIV screening in the United States: effect on clinical outcomes, HIV transmission, and costs. In *Annals of internal medicine* 145 (11), pp. 797–806. DOI: 10.7326/0003-4819-145-11-200612050-00004.

Paltiel, A. David; Weinstein, Milton C.; Kimmel, April D.; Seage, George R.; Losina, Elena; Zhang, Hong et al. (2005): Expanded screening for HIV in the United States--an analysis of cost-effectiveness. In *The New England journal of medicine* 352 (6), pp. 586–595. DOI: 10.1056/NEJMsa042088.

Pinkerton, S. D.; Holtgrave, D. R.; DiFranceisco, W.; Semaan, S.; Coyle, S. L.; Johnson-Masotti, A. P. (2000): Cost-threshold analyses of the National AIDS Demonstration Research HIV prevention interventions. In *AIDS (London, England)* 14 (9), pp. 1257–1268. DOI: 10.1097/00002030-200006160-00024.

Pinkerton, S. D.; Johnson-Masotti, A. P.; Otto-Salaj, L. L.; Stevenson, L. Y.; Hoffmann, R. G. (2001): Cost-effectiveness of an HIV prevention intervention for mentally ill adults. In *Mental health services research* 3 (1), pp. 45–55. DOI: 10.1023/a:1010112619165.

Pinkerton, Steven D.; Kibicho, Jennifer W.; Galletly, Carol L. (2013): Is the US AIDS Drug Assistance Program Cost-effective? In *AIDS and behavior* 17 (1), pp. 1–4. DOI: 10.1007/s10461-012-0321-z.

Pinkerton, Steven D.; Martin, Jeffrey N.; Roland, Michelle E.; Katz, Mitchell H.; Coates, Thomas J.; Kahn, James O. (2004): Cost-effectiveness of postexposure prophylaxis after sexual or injection-drug exposure to human immunodeficiency virus. In *Archives of internal medicine* 164 (1), pp. 46–54. DOI: 10.1001/archinte.164.1.46.

Pollack, H. A. (2001): Cost-effectiveness of harm reduction in preventing hepatitis C among injection drug users. In *Medical decision making : an international journal of the Society for Medical Decision Making* 21 (5), pp. 357–367. DOI: 10.1177/0272989X0102100502.

Popping, Stephanie; Hullegie, Sebastiaan J.; Boerekamps, Anne; Rijnders, Bart J. A.; Knegt, Robert J. de; Rockstroh, Jürgen K. et al. (2019): Early treatment of acute hepatitis C infection is cost-effective in HIV-infected men-who-have-sex-with-men. In *PloS one* 14 (1), e0210179. DOI: 10.1371/journal.pone.0210179.

Pottie, Kevin; Lotfi, Tamara; Kilzar, Lama; Howeiss, Pamela; Rizk, Nesrine; Akl, Elie A. et al. (2018): The Effectiveness and Cost-Effectiveness of Screening for HIV in Migrants in the EU/EEA: A Systematic Review. In *International journal of environmental research and public health* 15 (8). DOI: 10.3390/ijerph15081700.

Prabhu, Vimalanand S.; Farnham, Paul G.; Hutchinson, Angela B.; Soorapanth, Sada; Heffelfinger, James D.; Golden, Matthew R. et al. (2011): Cost-effectiveness of HIV screening in STD clinics, emergency departments, and inpatient units: a model-based analysis. In *PloS one* 6 (5), e19936. DOI: 10.1371/journal.pone.0019936.

Rahman, Mohammad M.; Khan, Mahmud; Gruber, DeAnn (2015): A Low-Cost Partner Notification Strategy for the Control of Sexually Transmitted Diseases: A Case Study From Louisiana. In *American journal of public health* 105 (8), pp. 1675–1680. DOI: 10.2105/AJPH.2014.302434.

Resch, Stephen; Altice, Frederick L.; Paltiel, A. David (2005): Cost-effectiveness of HIV screening for incarcerated pregnant women. In *Journal of acquired immune deficiency syndromes (1999)* 38 (2), pp. 163–173. DOI: 10.1097/01.qai.0000146598.40301.e6.

Ruger, Jennifer Prah; Abdallah, Arbi Ben; Ng, Nora Y.; Luekens, Craig; Cottler, Linda (2014): Cost-effectiveness of interventions to prevent HIV and STDs among women: a randomized controlled trial. In *AIDS and behavior* 18 (10), pp. 1913–1923. DOI: 10.1007/s10461-014-0745-8.

Sadler, Susannah; Tosh, Jon; Pennington, Rebekah; Rawdin, Andrew; Squires, Hazel; Romero, Carmen et al. (2017): A cost-effectiveness analysis of condom distribution programmes for the prevention of sexually transmitted infections in England. In *Journal of epidemiology and community health* 71 (9), pp. 897–904. DOI: 10.1136/jech-2017-209020.

Safren, Steven A.; Perry, Nicholas S.; Blashill, Aaron J.; O'Cleirigh, Conall; Mayer, Kenneth H. (2015): The cost and intensity of behavioral interventions to promote HIV treatment for prevention among HIV-positive men who have sex with men. In *Archives of sexual behavior* 44 (7), pp. 1833–1841. DOI: 10.1007/s10508-014-0455-3.

Sansom, Stephanie L.; Jamieson, Denise J.; Farnham, Paul G.; Bulterys, Marc; Fowler, Mary Glenn (2003): Human immunodeficiency virus retesting during pregnancy: costs and effectiveness in preventing perinatal transmission. In *Obstetrics and gynecology* 102 (4), pp. 782–790. DOI: 10.1016/s0029-7844(03)00624-0.

Schackman, Bruce R.; Eggman, Ashley A. (2012): Cost-effectiveness of pre-exposure prophylaxis for HIV: a review. In *Current opinion in HIV and AIDS* 7 (6), pp. 587–592. DOI: 10.1097/COH.0b013e3283582c8b.

Schackman, Bruce R.; Leff, Jared A.; Barter, Devra M.; DiLorenzo, Madeline A.; Feaster, Daniel J.; Metsch, Lisa R. et al. (2015): Cost-effectiveness of rapid hepatitis C virus (HCV) testing and simultaneous rapid HCV and HIV testing in substance abuse treatment programs. In *Addiction (Abingdon, England)* 110 (1), pp. 129–143. DOI: 10.1111/add.12754.

Schackman, Bruce R.; Metsch, Lisa R.; Colfax, Grant N.; Leff, Jared A.; Wong, Angela; Scott, Callie A. et al. (2013): The cost-effectiveness of rapid HIV testing in substance abuse treatment: results of a randomized trial. In *Drug and alcohol dependence* 128 (1-2), pp. 90–97. DOI: 10.1016/j.drugalcdep.2012.08.009.

Schneider, Karen; Gray, Richard T.; Wilson, David P. (2014): A cost-effectiveness analysis of HIV preexposure prophylaxis for men who have sex with men in Australia. In *Clinical infectious diseases : an official publication of the Infectious Diseases Society of America* 58 (7), pp. 1027–1034. DOI: 10.1093/cid/cit946.

Selvapatt, Nowlan; Ward, Thomas; Harrison, Lorna; Lombardini, Jody; Thursz, Mark; McEwan, Phil; Brown, Ashley (2017): The cost impact of outreach testing and treatment for hepatitis C in an urban Drug Treatment Unit. In *Liver international : official journal of the International Association for the Study of the Liver* 37 (3), pp. 345–353. DOI: 10.1111/liv.13240.

Shen, Mingwang; Xiao, Yanni; Rong, Libin; Meyers, Lauren Ancel; Bellan, Steven E. (2018): The cost-effectiveness of oral HIV pre-exposure prophylaxis and early antiretroviral therapy in the presence of drug resistance among men who have sex with men in San Francisco. In *BMC medicine* 16 (1), p. 58. DOI: 10.1186/s12916-018-1047-1.

Shepherd, J.; Kavanagh, J.; Picot, J.; Cooper, K.; Harden, A.; Barnett-Page, E. et al. (2010): The effectiveness and cost-effectiveness of behavioural interventions for the prevention of sexually transmitted infections in young people aged 13-19: a systematic review and economic evaluation. In *Health technology assessment (Winchester, England)* 14 (7), 1-206, iii-iv. DOI: 10.3310/hta14070.

Shrestha, Ram K.; Begley, Elin B.; Hutchinson, Angela B.; Sansom, Stephanie L.; Song, Binwei; Voorhees, Kelly et al. (2009): Costs and effectiveness of partner counseling and referral services with rapid testing for HIV in Colorado and Louisiana, United States. In *Sexually transmitted diseases* 36 (10), pp. 637–641. DOI: 10.1097/OLQ.0b013e3181a96d3d.

Shrestha, Ram K.; Clark, Hollie A.; Sansom, Stephanie L.; Song, Binwei; Buckendahl, Holly; Calhoun, Cindy B. et al. (2008): Cost-effectiveness of finding new HIV diagnoses using rapid HIV testing in community-based organizations. In *Public health reports (Washington, D.C. : 1974)* 123 Suppl 3, pp. 94–100. DOI: 10.1177/00333549081230S312.

Shrestha, Ram K.; Sansom, Stephanie L.; Kimbrough, Lisa; Hutchinson, Angela B.; Daltry, Daniel; Maldonado, Waleska et al. (2010): Cost-effectiveness of using social networks to identify undiagnosed HIV infection among minority populations. In *Journal of public health management and practice : JPHMP* 16 (5), pp. 457–464. DOI: 10.1097/PHH.0b013e3181cb433b.

Shrestha, Ram K.; Sansom, Stephanie L.; Schulden, Jeffrey D.; Song, Binwei; Smith, Linney C.; Ramirez, Ramon et al. (2011): Costs and effectiveness of finding new HIV diagnoses by using rapid testing in transgender communities. In *AIDS education and prevention : official publication of the International Society for AIDS Education* 23 (3 Suppl), pp. 49–57. DOI: 10.1521/aeap.2011.23.3_supp.49.

Song, Dahye L.; Altice, Frederick L.; Copenhaver, Michael M.; Long, Elisa F. (2015): Cost-effectiveness analysis of brief and expanded evidence-based risk reduction interventions for HIV-infected people who inject drugs in the United States. In *PloS one* 10 (2), e0116694. DOI: 10.1371/journal.pone.0116694.

Spaulding, Anne C.; Pinkerton, Steven D.; Superak, Hillary; Cunningham, Marc J.; Resch, Stephen; Jordan, Alison O.; Yang, Zhou (2013): Cost analysis of enhancing linkages to HIV care following jail: a cost-effective intervention. In *AIDS and behavior* 17 Suppl 2, S220-6. DOI: 10.1007/s10461-012-0353-4.

Suijkerbuijk, Anita W. M.; Over, Eelco A. B.; van Aar, Fleur; Götz, Hannelore M.; van Benthem, Birgit H. B.; Lugnér, Anna K. (2018a): Consequences of restricted STI testing for young heterosexuals in the Netherlands on test costs and QALY losses. In *Health policy (Amsterdam, Netherlands)* 122 (2), pp. 198–203. DOI: 10.1016/j.healthpol.2017.12.001.

Suijkerbuijk, Anita W. M.; van Hoek, Albert Jan; Koopsen, Jelle; Man, Robert A. de; Mangen, Marie-Josee J.; Melker, Hester E. de et al. (2018b): Cost-effectiveness of screening for chronic hepatitis B and C among migrant populations in a low endemic country. In *PloS one* 13 (11), e0207037. DOI: 10.1371/journal.pone.0207037.

Sweeney, Sedona; Ward, Zoe; Platt, Lucy; Guinness, Lorna; Hickman, Matthew; Hope, Vivian et al. (2019): Evaluating the cost-effectiveness of existing needle and syringe programmes in preventing hepatitis C transmission in people who inject drugs. In *Addiction (Abingdon, England)* 114 (3), pp. 560–570. DOI: 10.1111/add.14519.

Thanh, Nguyen X.; Akpinar, Ilke; Gratrix, Jennifer; Plitt, Sabrina; Smyczek, Petra; Read, Ron et al. (2017): Benefit of adjunct universal rectal screening for Chlamydia genital infections in women attending Canadian sexually transmitted infection clinics. In *International journal of STD & AIDS* 28 (13), pp. 1311–1324. DOI: 10.1177/0956462417704344.

Tuli, Karunesh; Kerndt, Peter R. (2009): Preventing sexually transmitted infections among incarcerated men who have sex with men: a cost-effectiveness analysis. In *Sexually transmitted diseases* 36 (2 Suppl), S41-8. DOI: 10.1097/OLQ.0b013e3181574daa.

Tuli, Karunesh; Sansom, Stephanie; Purcell, David W.; Metsch, Lisa R.; Latkin, Carl A.; Gourevitch, Marc N.; Gómez, Cynthia A. (2005): Economic evaluation of an HIV prevention intervention for seropositive injection drug users. In *Journal of public health management and practice : JPHMP* 11 (6), pp. 508–515. DOI: 10.1097/00124784-200511000-00006.

van de Vijver, David A. M. C.; Richter, Ann-Kathrin; Boucher, Charles A. B.; Gunsenheimer-Bartmeyer, Barbara; Kollan, Christian; Nichols, Brooke E. et al. (2019): Cost-effectiveness and budget effect of pre-exposure prophylaxis for HIV-1 prevention in Germany from 2018 to 2058. In *Euro surveillance : bulletin Europeen sur les maladies transmissibles = European communicable disease bulletin* 24 (7). DOI: 10.2807/1560-7917.ES.2019.24.7.1800398.

Varghese, B.; Peterman, T. A. (2001): Cost-effectiveness of HIV counseling and testing in US prisons. In *Journal of urban health : bulletin of the New York Academy of Medicine* 78 (2), pp. 304–312. DOI: 10.1093/jurban/78.2.304.

Venkatesh, Kartik K.; Lurie, Mark N.; Mayer, Kenneth H. (2010): How HIV treatment could result in effective prevention. In *Future virology* 5 (4), pp. 405–415. DOI: 10.2217/fvl.10.38.

Vriend, Henrike J.; Lugnér, Anna K.; Xiridou, Maria; van der Schim Loeff, Maarten F.; Prins, Maria; Vries, Henry J. C. de et al. (2013): Sexually transmitted infections screening at HIV treatment centers for MSM can be cost-effective. In *AIDS (London, England)* 27 (14), pp. 2281–2290. DOI: 10.1097/QAD.0b013e32836281ee.

Vries, Robin de; van Bergen, Jan E. A. M.; Jong-van den Berg, Lolkje T. W. de; Postma, Maarten J. (2008): Cost-utility of repeated screening for Chlamydia trachomatis. In *Value in health : the journal of the International Society for Pharmacoeconomics and Outcomes Research* 11 (2), pp. 272–274. DOI: 10.1111/j.1524-4733.2007.00225.x.

Walensky, Rochelle P.; Freedberg, Kenneth A.; Weinstein, Milton C.; Paltiel, A. David (2007): Cost-effectiveness of HIV testing and treatment in the United States. In *Clinical infectious diseases : an official publication of the Infectious Diseases Society of America* 45 Suppl 4, S248-54. DOI: 10.1086/522546.

Walensky, Rochelle P.; Weinstein, Milton C.; Kimmel, April D.; Seage, George R.; Losina, Elena; Sax, Paul E. et al. (2005): Routine human immunodeficiency virus testing: an economic evaluation of current guidelines. In *The American journal of medicine* 118 (3), pp. 292–300. DOI: 10.1016/j.amjmed.2004.07.055.

Wang, L. Y.; Davis, M.; Robin, L.; Collins, J.; Coyle, K.; Baumler, E. (2000): Economic evaluation of Safer Choices: a school-based human immunodeficiency virus, other sexually transmitted diseases, and pregnancy prevention program. In *Archives of pediatrics & adolescent medicine* 154 (10), pp. 1017–1024. DOI: 10.1001/archpedi.154.10.1017.

Wang, Li Yan; Burstein, Gale R.; Cohen, Deborah A. (2002): An economic evaluation of a school-based sexually transmitted disease screening program. In *Sexually transmitted diseases* 29 (12), pp. 737–745. DOI: 10.1097/00007435-200212000-00001.

Wilson, David; Fraser, Nicole (2014): Who pays and why? Costs, effectiveness, and feasibility of HIV treatment as prevention. In *Clinical infectious diseases : an official publication of the Infectious Diseases Society of America* 59 Suppl 1, S28-31. DOI: 10.1093/cid/ciu300.

Wilson, David P.; Heymer, Kelly-Jean; Anderson, Jonathan; O'Connor, Jody; Harcourt, Christine; Donovan, Basil (2010): Sex workers can be screened too often: a cost-effectiveness analysis in Victoria, Australia. In *Sexually transmitted infections* 86 (2), pp. 117–125. DOI: 10.1136/sti.2009.036673.

Wit, G. Ardine de; Over, Eelco A. B.; Schmid, Boris V.; van Bergen, Jan E. A. M.; van den Broek, Ingrid V. F.; van der Sande, Marianne A. B. et al. (2015): Chlamydia screening is not cost-effective at low participation rates: evidence from a repeated register-based implementation study in The Netherlands. In *Sexually transmitted infections* 91 (6), pp. 423–429. DOI: 10.1136/sextrans-2014-051677.

Wodak, Alex; Cooney, Annie (2005): Effectiveness of sterile needle and syringe programmes. In *International Journal of Drug Policy* 16, pp. 31–44. DOI: 10.1016/j.drugpo.2005.02.004.

Yazdanpanah, Yazdan; Perelman, Julian; DiLorenzo, Madeline A.; Alves, Joana; Barros, Henrique; Mateus, Céu et al. (2013): Routine HIV screening in Portugal: clinical impact and cost-effectiveness. In *PloS one* 8 (12), e84173. DOI: 10.1371/journal.pone.0084173.

Zaric, Gregory S.; Bayoumi, Ahmed M.; Brandeau, Margaret L.; Owens, Douglas K. (2008): The cost-effectiveness of counseling strategies to improve adherence to highly active antiretroviral therapy among men who have sex with men. In *Medical decision making : an international journal of the Society for Medical Decision Making* 28 (3), pp. 359–376. DOI: 10.1177/0272989X07312714.

Zulliger, Rose; Maulsby, Cathy; Solomon, Liza; Baytop, Chanza; Orr, Alex; Nasrullah, Muazzam et al. (2017): Cost-utility of HIV Testing Programs Among Men Who Have Sex with Men in the United States. In *AIDS and behavior* 21 (3), pp. 619–625. DOI: 10.1007/s10461-016-1547-y.

Zwart, Jolijn M.; Mangen, Marie-Josee J.; Bartelsman, Menne; van Rooijen, Martijn S.; Vries, Henry J. C. de; Xiridou, Maria (2019): Microscopic examination of Gram-stained smears for anogenital gonorrhoea in men who have sex with men is cost-effective: evidence from a modelling study. In *Sexually transmitted infections* 95 (1), pp. 13–20. DOI: 10.1136/sextrans-2018-053578.
